# Non-Circadian Expression Masking Clock-Driven Weak Transcription Rhythms in U2OS Cells

**DOI:** 10.1371/journal.pone.0102238

**Published:** 2014-07-09

**Authors:** Julia Hoffmann, Laura Symul, Anton Shostak, Tamás Fischer, Felix Naef, Michael Brunner

**Affiliations:** 1 Biochemistry Center, University of Heidelberg, Heidelberg, Germany; 2 Institute of Bioengineering, École Polytechnique Fédérale de Lausanne, Lausanne, Switzerland; McGill University, Canada

## Abstract

U2OS cells harbor a circadian clock but express only a few rhythmic genes in constant conditions. We identified 3040 binding sites of the circadian regulators BMAL1, CLOCK and CRY1 in the U2OS genome. Most binding sites even in promoters do not correlate with detectable rhythmic transcript levels. Luciferase fusions reveal that the circadian clock supports robust but low amplitude transcription rhythms of representative promoters. However, rhythmic transcription of these potentially clock-controlled genes is masked by non-circadian transcription that overwrites the weaker contribution of the clock in constant conditions. Our data suggest that U2OS cells harbor an intrinsically rather weak circadian oscillator. The oscillator has the potential to regulate a large number of genes. The contribution of circadian versus non-circadian transcription is dependent on the metabolic state of the cell and may determine the apparent complexity of the circadian transcriptome.

## Introduction

Circadian clocks are self-sustained oscillators that depend on interlocked transcriptional feedback loops. In mammals, the core loop consists of the transcriptional activators BMAL1 and CLOCK, which dimerize, bind to E-box elements, and activate transcription of Cryptochrome (*CRY1* and *CRY2*) and Period (*PER1*, *PER2*, and *PER3*) genes [1], [2]. CRY and PER proteins are repressors inhibiting the activity of BMAL1/CLOCK [3], [4]. In a second loop ROR activators and REV-ERB repressors regulate rhythmic transcription of *BMAL1* [5]–[7] and *NPAS2* [8] and presumably also *CRY* genes [7], [9] by competing for ROR elements [10].

The central circadian pacemaker resides in the suprachiasmatic nucleus (SCN). It is entrained by the geophysical day/night cycle of the earth’s rotation through light input received via the retinohypothalamic tract [11]. Many organs and cell types also contain a circadian clock [12]–[15]. These peripheral clocks are synchronized by the SCN [16] and, in addition, by rhythmic cues independent of the SCN, such as entrainment by rhythmic feeding in case of the hepatic clock [17].

Circadian clocks have the potential to drive rhythmic expression of a large number of clock-controlled genes on a transcriptional and posttranscriptional level [18]–[21], which is crucial for circadian rhythms in physiology and behavior.

In mouse liver, an organ with a highly circadian physiology, up to ∼3700 transcripts are expressed in circadian fashion [19], [22]–. The rhythmic transcript levels of the core clock genes are controlled on the level of transcription while abundance rhythms of more than half of the clock-controlled transcripts may be based on post-transcriptional regulation [19], [20], [25]. ChIP-seq analyses indicate that the core circadian transcription regulators bind in circadian fashion to several thousand sites in the mouse liver genome [19], [23]. The vast majority of these binding sites cannot be associated with rhythmic gene transcription, the strongest sites, however, were highly predictive of rhythmic transcription [23].

In other tissues up to ∼10% of the transcriptome is expressed in circadian fashion but the subsets of rhythmic genes in different tissues and cell types show little overlap, suggesting tissue-specific regulation of circadian gene expression [18]. The molecular basis of tissue specific circadian abundance rhythms of expressed genes is not understood.

The human osteosarcoma cell line U2OS expresses circadian clock components that drive oscillation of core clock genes [26], [27]. However, it came as a surprise that besides seven rhythmic genes encoding core circadian clock components no further rhythmic transcripts had been detected in U2OS cells in a stringent genome-wide transcriptome analysis [22]. Thus, despite the presence of a circadian clock in liver and U2OS cells, the complexity of the circadian transcriptomes appear to be substantially different. We therefore asked, whether the circadian transcription factors bind to and regulate fewer genes in U2OS than in liver. We show that BMAL1, CLOCK, and the circadian repressor CRY1 bind to about 3000 sites in the genome of U2OS cells, a number similar to the binding sites identified in mouse liver [19], [23]. However, our own transcriptome analysis showed that only 58 genes with binding sites are rhythmically expressed, among these 11 genes encoding core clock components. The majority of promoters with binding sites do not support rhythmic accumulation of transcripts. Luciferase fusions with such promoters revealed low amplitude but robust transcription rhythms that persisted in constant conditions in essentially undamped manner for several days. However, non-circadian transcription activities overwrite the rhythmic contribution of the circadian clock. Inhibition of metabolism reduced the clock-dependent and/or independent contribution to transcription in promoter-specific manner. Our data suggests that isolated U2OS cells harbor a robust and precise clock. This clock has the potential to regulate a large number of genes in rhythmic fashion and with considerable amplitude. The relative activities of circadian versus gene-specific transcription regulators determine to the apparent complexity of the circadian transcriptome.

## Results

### Circadian regulator binding sites

**Figure 1:**
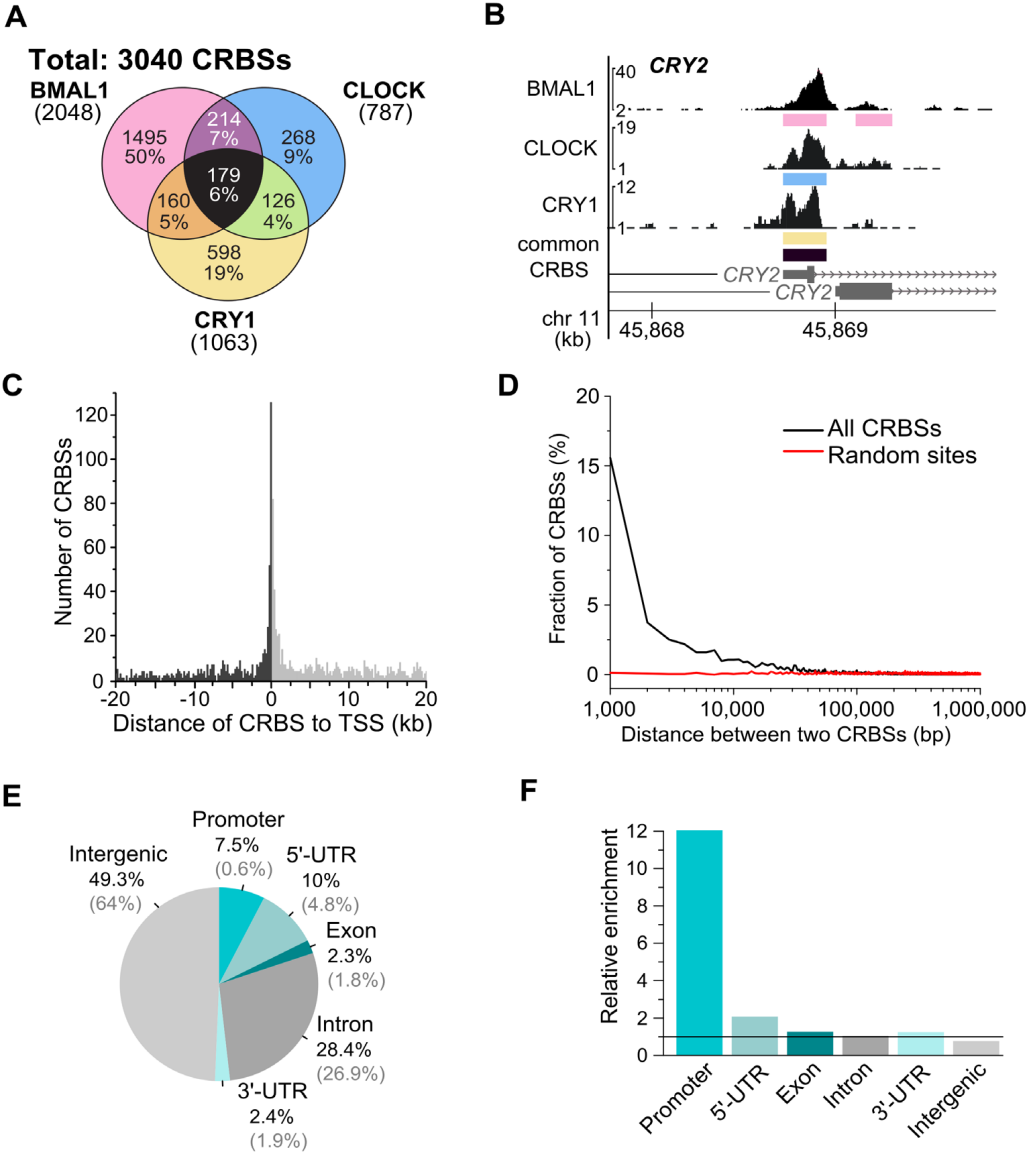
Genome-wide binding sites of the circadian transcription regulators BMAL1 CLOCK and CRY1. (**A**) Venn diagram showing numbers and percentage of individual and overlapping binding sites of BMAL1, CLOCK, and CRY1. Percentages represent the fraction of the CRBSs over the total number of sites for the 3 proteins (**B**) UCSC browser views of BMAL1 (top), CLOCK (middle) and CRY1 (bottom) occupancy at the promoter of *CRY2*. Regions detected as binding sites of the individual transcription regulators are indicated by colored bars. Black bars indicate common binding sites of the three regulators. (**C**) CRBSs cluster near TSSs. Histograms of positions of all CRBSs are shown for a window of ±20 kb around TSSs with a bin size of 200 bp. 352 CRBSs are enriched ±1 kb around TSS. (**D**) Histogram of the distance between two consecutive CRBSs (black) in comparison to a set of 3040 random sites (red). (**E**) Pie chart showing the percentage of binding sites in a genomic region (black) and the contribution of the region (%) to the genome (grey). (**F**) Genomic annotation of the CRBSs to promoter (−1 kb to TSS), 5′UTR, exon, intron, 3′UTR, and intergenic region.

We selected the immortalized human osteosarcoma cell line U2OS to identify and characterize binding sites of the circadian transcription factor CLOCK/BMAL1 and its inhibitor CRY1 by chromatin immunoprecipitation and next generation sequencing (ChIP-seq). Using affinity purified antibodies (Figure S1A) we identified 3040 circadian regulator binding sites (CRBSs) in the genome of unsynchronized U2OS cells (Figure 1A). Examples of CRBSs in the *CRY2* promoter are shown in Figure 1B. A circadian ChIP-PCR profile of BMAL1 at the promoters of PER2 and REV/ERBa revealed that the transcription factor interacts rhythmically and with high amplitude with the CRBSs (Figure S1B).

The CRBSs were highly enriched at transcription start sites (TSSs) (Figure 1C). The median distance between CRBSs was 47 kb (Figure 1D), indicating that the sites were not randomly distributed in the genome. The majority of the CRBSs was located in intergenic regions (49.3%) and introns (28.4%), and 7.5% of the CRBSs were in promoters (Figure 1E). Taking in consideration that intergenic regions and introns make up 64.1% and 27.1% of the genome, respectively, CRBSs were highly enriched in promoters (Figure 1F).

Sequence analysis of the CRBSs revealed an enrichment of E-box motifs, in particular CACGTG, CA**T**GTG and CA**G**GTG, and tandem E-boxes with a 6 bp spacer (Figures S2), in agreement with previous data from mouse liver [23].

Recently it has been reported that CRYs also interact with the glucocorticoid receptor [28]. Glucocorticoid receptor response elements (GREs) were not enriched in the CRY1 binding sites suggesting that interaction with the glucocorticoid receptor may not be a major pathway of CRY1 recruitment to chromatin in U2OS cells.

### Rhythmic gene expression

Each CRBS was attributed to the gene with the closest TSS using the RefSeq gene annotation. By this means we defined 1373 genes with binding sites (Dataset S1).

We then analyzed the temporal expression profiles of genes with CRBSs. For control we analyzed expression of a randomly chosen set of 3480 genes (random genes). In addition, we selected and analyzed 1503 genes that are implied in different cellular pathways, including the circadian clock genes *CLOCK, BMAL1, E4BP4, and NPAS2*.

**Figure 2:**
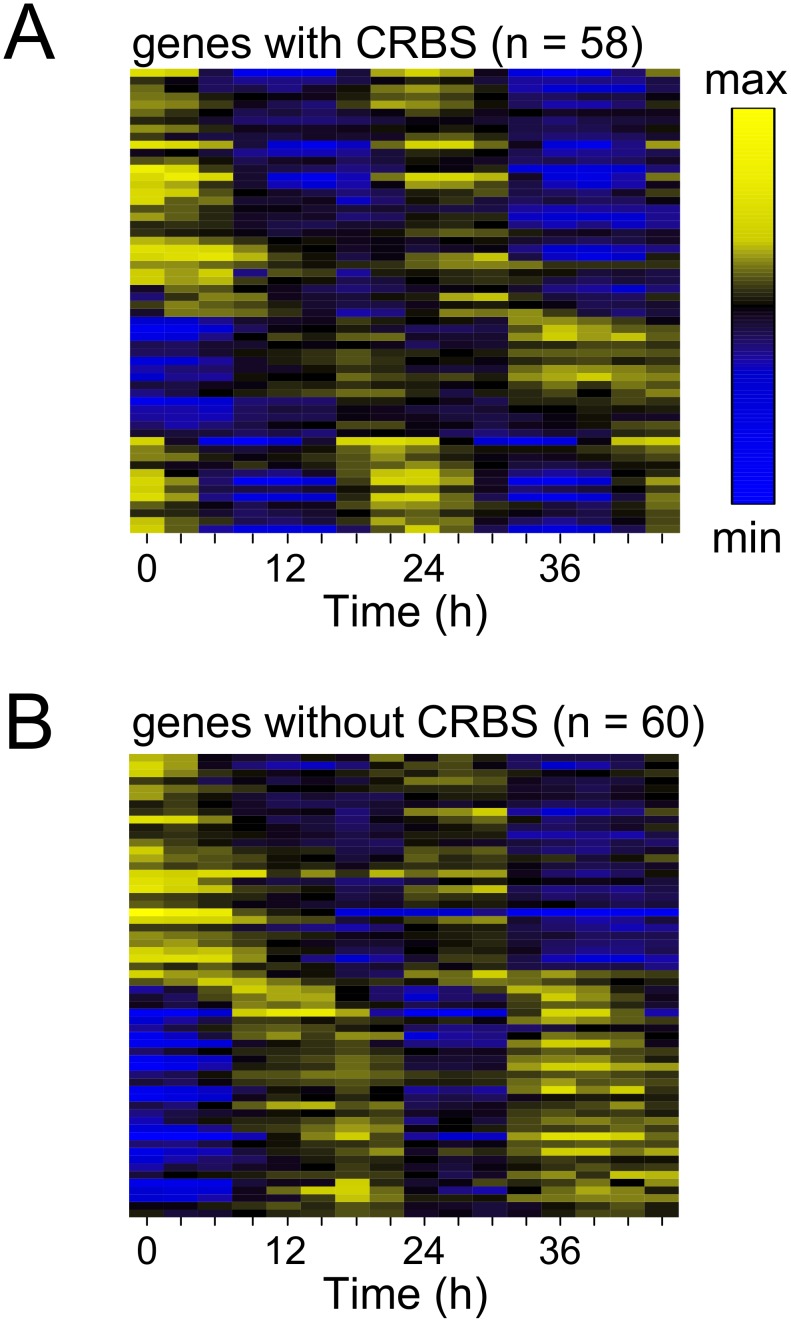
Heat map view of 24 h cycling genes. Rhythmic genes with CRBSs (**A**) and without CRBSs (**B**) were ordered by phase of expression. Temperature entrained U2OS cells were released into constant conditions (37°C) and RNA expression levels were analyzed in 3 h intervals over a period of two days.

**Figure 3:**
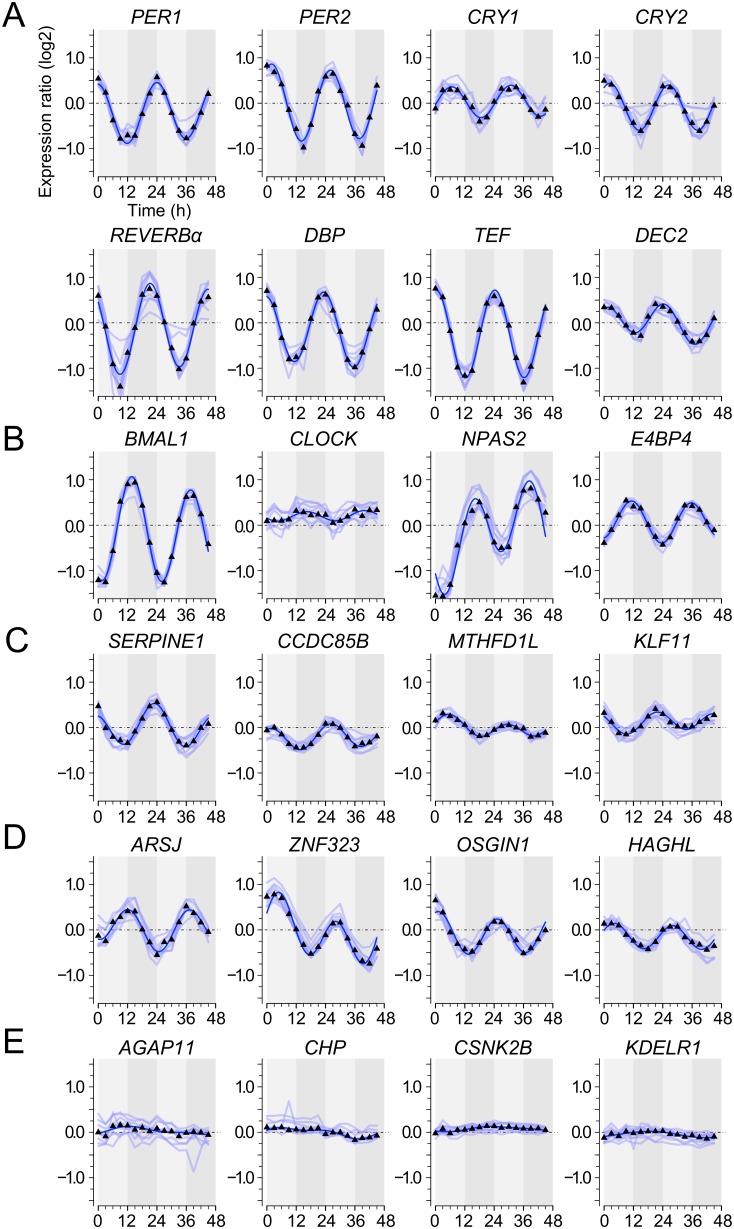
Temporal expression profiles of rhythmic and non-rhythmic genes. RNA levels of U2OS cells were analyzed in 3(0–45 h) relative to the average were calculated and plotted on a log2 scale versus the circadian time (CT). For each gene up to 10 probes were spotted on the arrays. Light blue lines correspond to expression profiles based on individual probes. The black triangles and the fitted dark blue sine curve correspond to the median of the data. Light and dark areas in the background indicate subjective day and night, respectively. Examples are shown for genes that fall in various categories. (**A**) Clock genes with CRBSs. (**B**) Clock-genes without CRBSs. (**C**) Rhythmic genes with CRBSs. (**D**) Rhythmic genes without CRBSs. (**E**) Genes not expressed in a rhythmic fashion. *AGAP11* harbors a highly enriched binding site for BMAL1, CLOCK, and CRY1, *CHP* has a CRY1 binding site. In both genes the CRBSs were close to the TSS. *CSNK2B* and *KDELR1* do not have a CRBS in a window of ±20 kb.

U2OS cells were entrained for 5 days with temperature cycles (12 h, 33°C/12 h, 37°C) and then released to constant conditions (37°C). Samples were harvested at three-hour intervals for two consecutive days. We performed two independent experiments using two entrainment protocols (Materials and methods). 58 genes with CRBSs, and 60 genes without CRBSs were rhythmically expressed in both experiments (Figure 2, Figure S3). The proportion of rhythmic genes with a CRBS for any of the regulators was similar to the proportion of rhythmic genes with CRBSs for all regulators. By extrapolation, we estimate that about 0.9–1.2% of the genes are rhythmically expressed in U2OS cells. Amongst the rhythmic genes with CRBSs were the circadian genes *CRY1, CRY2, DEC1, DEC2, DBP, PER1, PER2, PER3, REVERBα, REVERBβ* and *TEF*, which are regulated by BMAL1/CLOCK (Figure 3A and S4A). In the group of rhythmic genes without CRBSs were *BMAL1, E4BP4, and NPAS2* (Figure 3B and S4B). Thus, virtually all known rhythmic clock genes were detected, indicating that the expression analysis was suitable for the detection of circadian genes. Expression rhythms of these core clock genes oscillated with about 4-fold amplitude (peak: trough). The *CLOCK* gene did not qualify as a rhythmic gene by our criteria (Figure 3B). Examples of rhythmic genes with and without CRBSs and non-rhythmic genes are shown in Figure 3C–E and Figures S3 and S4C–E.

**Figure 4:**
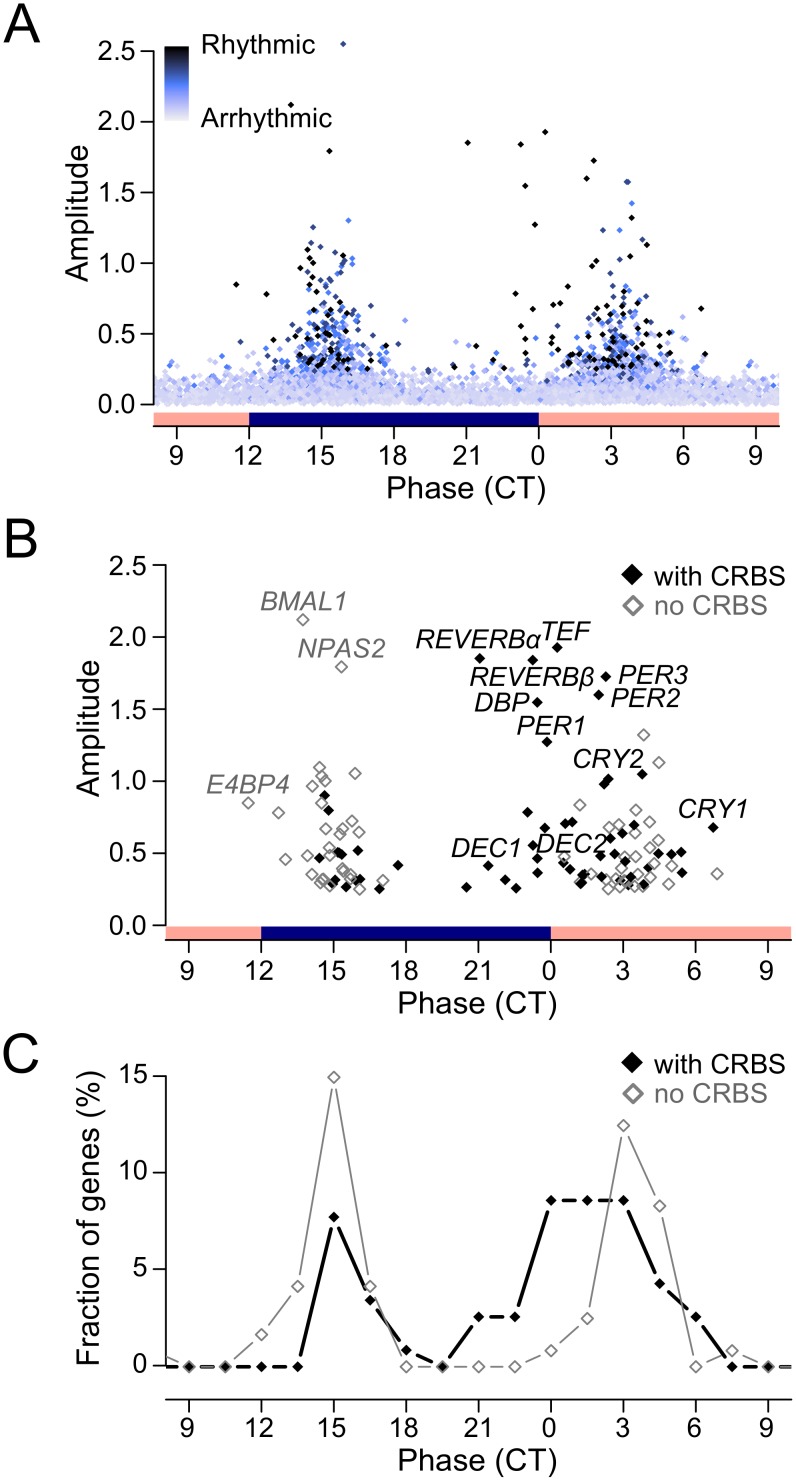
mRNA expression phase and correlations with CRBSs. Analysis of temporal expression profiles of 5708 expressed genes in two microarray replicates identified 118 common genes with circadian expression rhythms. Data from array A is shown. (**A**) The amplitudes of expressed genes (n = 5708) were plotted versus the circadian phase. The 118 rhythmic genes are indicated by black symbols. The shade of blue corresponds to the rhythmicity of a gene (light blue = low 24 h-rhythmicity and/or phase undefined; dark blue = high 24 h-rhythmicity and highly reliable phase). (**B**) The amplitudes of the 118 rhythmic genes are plotted against the phase. Genes with CRBSs are shown with black diamonds, the other rhythmic genes are displayed with gray diamonds. Core circadian clock genes are indicated. (**C**) Phase distribution of rhythmic genes with and without CRBSs.

A plot of the amplitude versus the circadian phase revealed that rhythmic genes were expressed at two phases (∼CT15 and ∼CT3) and displayed generally rather low amplitude rhythms (Figure 4A). The peak at ∼CT3 contained all known core clock genes that are controlled by BMAL1/CLOCK (Figure 4B and C). Corresponding data for array B are shown in Figure S5.

We then divided the group of genes with CRBSs (n = 1373) into two categories, genes with at least one CRBS close to the TSS (±1 kb, n = 352) or only distant CRBSs (n = 1021). The fraction of rhythmic genes with distant CRBSs was 3.2%, while 7.9% of the genes with a CRBS close to the TSS were rhythmic (Figure S6, p<0.001). Thus, rhythmic gene expression correlates with the proximity of CRBSs to the TSS. Yet, most genes with CRBSs, even genes with highly enriched binding sites in their promoters, appeared not to be rhythmically expressed in U2OS cells.

In summary, U2OS cells express only a few rhythmic genes (∼1%). Core clock genes are generally expressed with readily detectable rhythms. Yet, the amplitudes (∼4x) are much lower than corresponding amplitudes in liver of living mice [19], [22], [24], [25].

### Circadian and non-circadian contributions to gene expression

**Figure 5:**
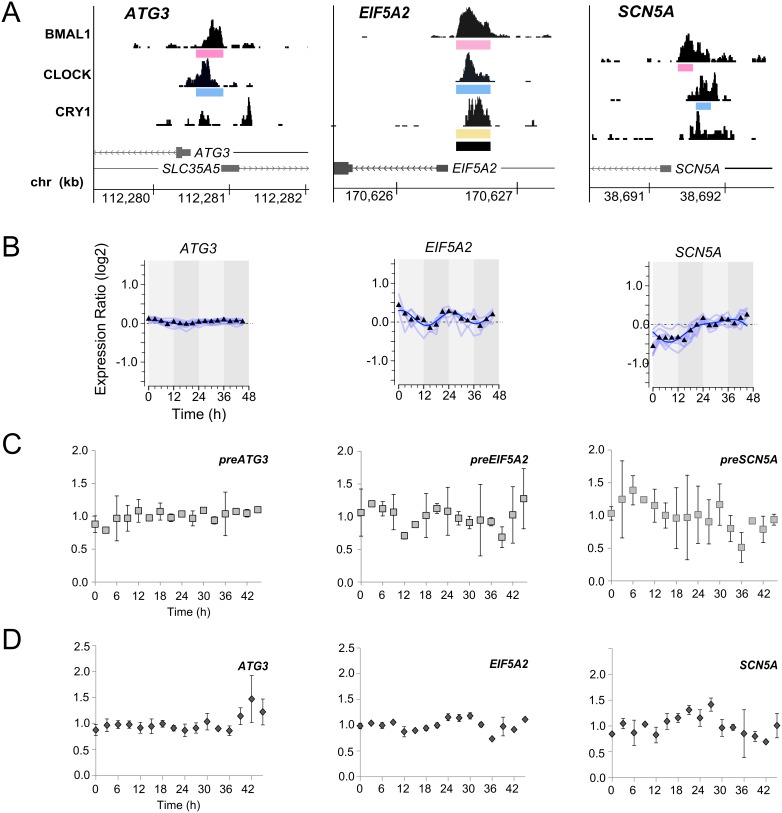
Expression analysis of genes with CRBSs in their promoters. (**A**) UCSC browser views of BMAL1 (top), CLOCK (middle) and CRY1 (bottom) occupancy at the promoters of *ATG3*, *EIF5A2*, and *SCN5A*. Binding sites of the individual transcription regulators are indicated by colored bars. Black bars indicate common binding sites of the three regulators. (**B**) Temporal expression profiles (microarray analysis) of *ATG3*, *EIF5A2*, and *SCN5A*, which have strong CRBSs in their promoters. (**C, D**) Around the clock qRT-PCR analysis of preRNA (**C**) and mRNA (**D**) levels of *ATG3*, *EIF5A2*, and *SCN5A*. qRT-PCR analysis was carried out with intron- and exon-specific probes, respectively, of *ATG3*, *EIF5A2,* and *SCN5A* using RNA of time courses A and B. Expression levels of *HPRT1* were used for normalization.

The promoters of *ATG3*, *EIFA2,* and *SCN5A* contain highly enriched binding sites close to their TSSs (Figure 5A) but did not qualify in the expression array analysis as rhythmic genes by our criteria (Figure 5B). preRNA and mRNA profiles (Figure 5C and D) indicate that *ATG3, EIF5A2* and *SCN5A* are not transcribed with a significant rhythm in U2OS cells. In contrast to U2OS cells, the strongest binding sites in mouse liver correlate well with rhythmic transcript levels [19], [23].

**Figure 6:**
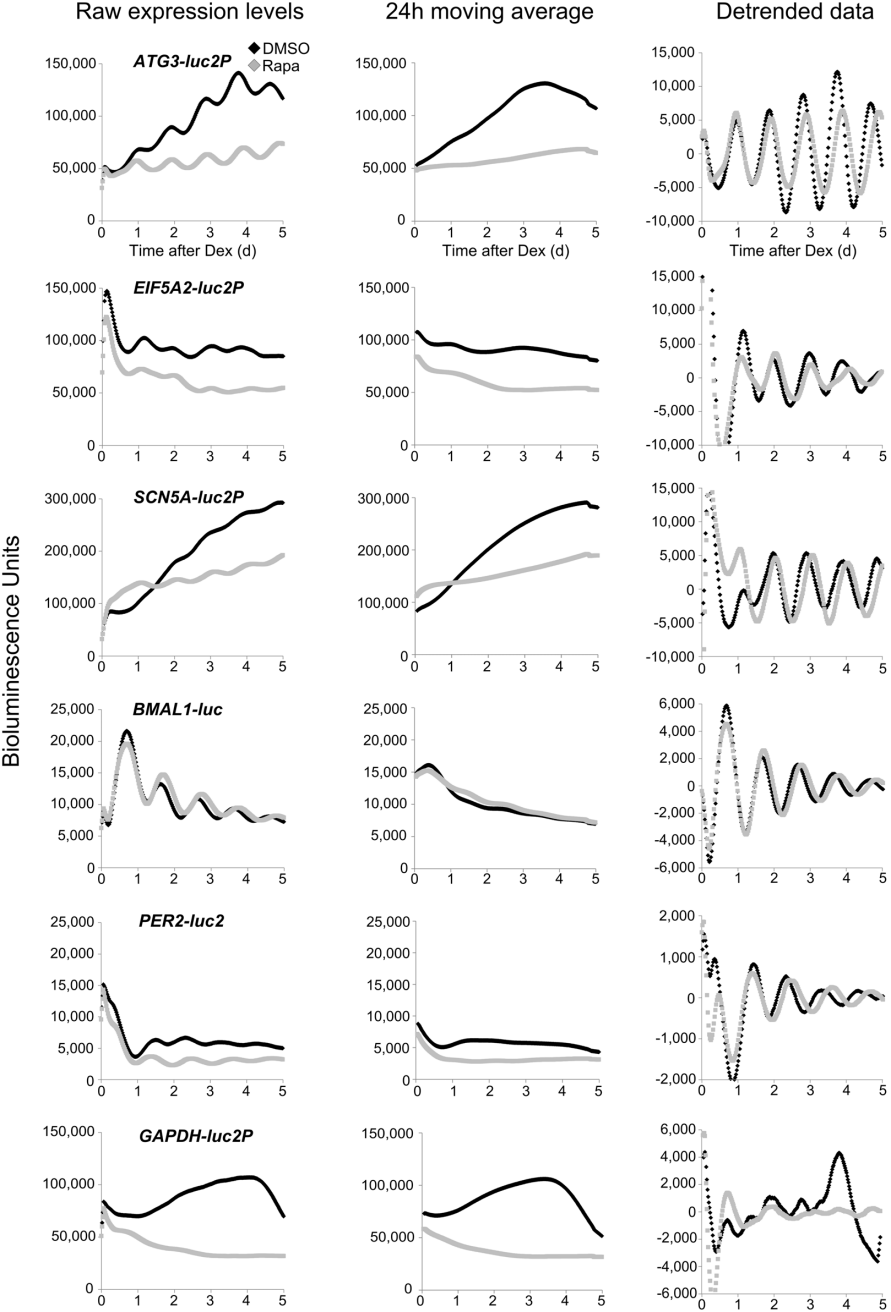
Rhythmic transcription of genes with CRBSs in their promoter is masked by high baseline expression. Robust low amplitude transcription rhythms revealed by long-term luminescence measurements of stable U2OS cell lines expressing the destabilized luciferase2P (*luc2P*) under control of the promoters of *ATG3*, *EIF5A2*, and *SCN5A*. *BMAL1*-*luc*, *PER2-luc2,* and *GAPDH-luc2P* cell lines are shown for control. Luminescence of synchronized cultures was measured at 30 min intervals. Raw expression data of the indicated reporter genes for day 1 to day 5 after synchronization are shown in the left panels. The middle panels show the 24 h moving average of the luciferase activity to estimate the non-rhythmic contribution and the right panels show the de-trended data to estimate the rhythmic contribution. The black and grey traces show expression profiles in the absence and presence of 200 nM rapamycin, respectively.

We generated stable reporter cell lines expressing a PEST-destabilized luciferase (LUC2P) under control of the promoters of *ATG3*, *EIF5A2*, *SCN5A and GAPDH* (promoter with no CRBS) and analyzed luciferase activity in comparison with *BMAL-luc* and *PER2-luc2* reporter cell lines. The raw data (Figure 6, left) revealed that the promoters of *ATG3*, *EIF5A2* and *SCN5A* supported, like *BMAL1 and PER2,* rhythmic luciferase expression. The temporal expression profiles were composed of an apparent non-circadian and a circadian component (Figure 6 middle and right). The non-circadian contributions to the temporal transcription profiles (24 h moving average) were promoter-specific and hence not entirely due to desychronization of cells. The non-circadian component was at all times stronger than the circadian component, resulting in severely blunted expression rhythms of *ATG3*, *EIF5A2* and *SCN5A*. The relative amplitudes (peak: trough) of the circadian transcription rhythms of the *ATG3*, *EIF5A2* and *SCN5A* reporters were low. Hence, expression of these clock-controlled reporter genes was apparently arrhythmic despite absolute amplitudes (peak – trough) similar or higher than those supported by *BMAL1-luc* and *PER2-luc2.* In contrast, the non-circadian contributions to *luciferase* expression by *BMAL1* and to a lesser extent by *PER2* was rather low and therefore these genes displayed circadian oscillations with significant amplitudes (peak: trough). Interestingly, the luciferase rhythms of *ATG3*, *EIF5A2* and *SCN5A* did not substantially dampen while the rhythms of *BMAL1* and *PER2* damped extensively during the same time period. The data suggests that the high amplitudes of the BMAL1 and PER2 oscillations were evoked by the synchronizing dexamethasone pulse given at the beginning of the measurement. *GAPDH-luc2P* expression was essentially arrhythmic.

The non-circadian components of the temporal expression profiles (Figure 6, middle panels) might be driven by promoter-specific transcription regulators. Such factors could respond in complex fashion to the temporally changing conditions in the 96-well plates that may challenge metabolism and cellular homeostasis. Since we have no means of changing the non-circadian versus circadian contribution to the expression of these genes in a specific manner we attempted to globally unbalance transcription. Rapamycin is a potent inhibitor of mTORC1 that regulates many aspects of metabolism [29] and may directly and indirectly affect expression of many genes. Hence, we incubated the reporter cell lines with rapamycin. The treatment did not significantly affect growth of the U2OS cells (Figure S7) but lengthened the circadian period by about ∼1.5 h (Figure 6, grey versus black traces). Rapamycin reduced the average expression levels of the *PER2, ATG3*, *EIF5A2 SCN5A* and *GAPDH* reporters but had no effect on the *BMAL1-luc* expression level and the amplitude of the rhythm (Figure 6). Rapamycin generally stabilized the temporal luciferase expression profiles of the reporters but had little or no effect on the absolute amplitudes (peak – trough) of the transcription rhythms. Accordingly, the relative amplitudes (peak: trough) supported by the promoters of *ATG3*, *EIF5A2 SCN5A* and *PER2* increased in the presence of rapamycin. The data suggest that these promoters are regulated by circadian and non-circadian factors, which respond in promoter-specific manner to the inhibition of mTORC1.

Together, the data suggests that promoters with binding sites for BMAL1/CLOCK support in principle rhythmic transcription. The rhythmic accumulation of transcript levels appears to be dependent on the activity of the circadian transcription factors versus genes specific, non-circadian transcription regulators. The data are compatible with the notion that U2OS cells harbor a rather weak circadian oscillator. This oscillator supports transcription rhythms with low amplitudes relative to the non-circadian expression levels of the clock-controlled genes.

## Discussion

Circadian oscillators in different cell types are composed of essentially identical components. Yet, the repertoires of clock-controlled genes as well as their expression amplitudes can differ between cell types or organs. We have correlated binding of circadian transcription regulators to the genome of U2OS cells with circadian expression rhythms of genes with such binding sites. The circadian regulators BMAL1, CLOCK, and CRY1 bound to several thousand sites (3040) in the U2OS genome. However, only 58 genes with CRBSs (including 11 core clock genes) were rhythmically expressed with significant, yet low amplitudes. Based on the analysis of a set of randomly chosen genes we extrapolate that U2OS cells may express about 300 genes in rhythmic fashion with detectable amplitudes. Our data are in principle consistent with data of a previous genome-wide expression analysis of U2OS cells, reporting that only the transcript levels of seven core clock genes oscillate in circadian fashion with significant amplitudes [22]. Our study indicates that U2OS cells rhythmically express core clock genes and in addition a rather small number of clock-controlled genes that oscillate with very low amplitudes.

The number of cycling transcripts in mouse liver is about an order of magnitude higher than in U2OS cells and levels of most clock-controlled liver transcripts oscillate with higher amplitude [19], [22], [24], [25], [30]. Also the SCN expresses *ccgs* in rhythmic fashion and with high amplitudes [18]. Why is the number of apparently rhythmic transcripts so low in U2OS cells?

We show that BMAL1 and CLOCK sites were highly enriched at TSSs, although to a somewhat lesser extent than in mouse liver [19], [23]. Yet, not even genes with strong CRBSs in their promoter displayed detectable circadian expression rhythms in constant conditions. Luciferase gene fusions of promoters with CRBSs revealed that the corresponding reporter genes were in fact expressed in circadian fashion. Since the promoter-luciferase fusion genes express approximately identical mRNAs the luciferase activity rhythms should essentially reflect transcription independent of posttranscriptional processes. It is therefore highly likely that promoters with CRBSs are rhythmically transcribed in U2OS cells. Given the number of CRBSs and their enrichment in promoters, U2OS cells may support rhythmic transcription of a similarly large number of genes as liver or the SCN. However, the amplitudes of the luciferase rhythms (peak: trough) were low in relation to the mean expression levels of the reporter genes. Low amplitude expression rhythms may however not be detected with statistical significance in genome-wide approaches like expression arrays.

Why are the amplitudes of rhythmic transcript levels low in U2OS cells and high in mouse liver *in* *vivo*? Reasons might be found on the level of a single cell and/or on the level of the population of cells. On the level of an individual cell, the strength or capacity of the circadian oscillator may depend in complex manner on expression levels and ratios of core and accessory clock components. The expression levels and amplitudes of the core clock genes are lower in U2OS cells than in liver [19], [22], [24], [25], [30], suggesting that U2OS cells may harbor a rather weak circadian oscillator, which may not have the capacity to support high amplitude rhythms of *ccgs*. On the systemic level, the dexamethasone pulse may only inefficiently synchronize the population of U2OS cells, which could explain the low amplitude circadian expression rhythms. However, it should be noted that the substantial dampening of the *BMAL1-luc* and *PER2-luc* rhythms after synchronization of the U2OS cells with dexamethasone does not reflect desynchronization of cells since the oscillations of the *ATG3*, *EIF5A2, and SCN5A* reporters rhythms persist during the same time period in essentially undamped manner. Furthermore, when cells desynchronize, the oscillation of *BMAL1-luc* and *PER2-luc* should dampen around a constant mean expression level, which is not the case. Rather, the initially high peak expression levels of *BMAL1-luc* and *PER2-luc* rhythms appear to be directly triggered by the dexamethasone pulse given at the beginning of the recording period. In mouse, several clock genes, including *Bmal1* and *Per2* respond to the glucocorticoid receptor [31]. The dexamethasone pulse induced transcription of *BMAL1-luc* and *PER2-luc* at levels that are higher than the expression levels supported by the circadian clock in constant conditions. The dampening of the *BMAL1-luc* and *PER2-luc* rhythms in the course of several days may reflect aftereffects of the dexamethasone pulse. At day one, dexamethasone induced expression of *EIF5A2* and *PER2* in a similar manner but did not affect expression of *ATG3* and *SCN5A*, suggesting that entraining cues may directly induce transcription of core clock genes and also of specific *ccgs*.

A weak oscillator in U2OS cells can only in part explain the low number of robustly cycling genes in U2OS cells since the U2OS clock is sufficiently strong to support transcription rhythms of the *ATG3*, *EIF5A2, and SCN5A* reporters with absolute amplitudes (peak – trough) equal or higher than those of the *BMAL1* and *PER2* promoters. Since the relative amplitudes (peak: trough) of the *ATG3*, *EIF5A2, and SCN5A* reporters are low, our data suggest that transcription of the *ccgs* is supported by additional, clock-independent factors. These components determine transcription levels in promoter-specific manner and mask the weaker contribution of the circadian clock.

It seems therefore conceivable that the apparent complexity of the rhythmic transcriptome of a cell is dependent on the strength of the circadian clock (e.g. expression levels and ratios of clock components) in comparison to the activity of non-circadian transcription factors regulating gene expression according to acute environmental conditions and in cell-type specific manner.

## Materials and Methods

### Antibodies

For all ChIPs, polyclonal anti-rabbit antibodies were raised by Pineda Antikörper-Service, Berlin, Germany and purified using standard techniques. BMAL1 was raised against a C-terminal peptide CLEADAGLGGPVDFSDLPWPL [23], CRY1 against the C-terminal peptide CQEEDTQSIGPKVQRQSTN and CLOCK against the N-terminal peptide CIFDGLVEEDDKDKAKRVS.

### ChIP protocol

Synchronized U2OS cells were fixed in 1% formaldehyde for 10 minutes followed by quenching in 125 mM glycine. After scraping cell were washed several times with PBS and then washed twice with cold IP buffer (50 mM Tris, pH 7.5, 150 mM NaCl, 5 mM EDTA, 0.5% NP40, 0.1% triton-X 100). Chromatin was sonicated using a Bioruptor (Diagenode Inc.) to obtain 200–1000 bp DNA fragments (30 sec on, 30 sec off cycles for 20 min on high power). Samples were incubated overnight with BMAL1 antibody followed by 2 hr incubation with protein A agarose beads (Millipore). After several washes with IP buffer, samples were boiled for 10 min in 10% Chelex (Bio-Rad) with Proteinase K (150 µg/mL) and spinned down. DNA-containing supernatant was collected for qPCR and values were normalized to percentage of input. Circadian analysis was performed using CircWave software.

Chromatin Immunoprecipitation for ChIP-sequencing was performed as described previously [23] using confluent desynchronized U2OS cells and BMAL1, CLOCK, and CRY1-antibodies. Immunoprecipitated chromatin (10 ng DNA of 8 independent BMAL1 ChIPs with enrichment levels >80-fold, 9 ng DNA of a CRY1 ChIP with 10-fold enrichment, and 8 ng DNA of a CLOCK ChIP with 40-fold enrichment) was used for library preparation with the ChIP-Seq Sample Preparation Kit (Illumina) and samples were quantified using a Qubit fluorometer (Invitrogen). Three lanes of the BMAL1 library, and one lane each of the CRY1 and CLOCK library were sequenced on the Illumina Genome Analyzer IIx using Single-Read Cluster Generation Kit and 36 Cycle Sequencing Kit v2 (Lausanne Genomics Technologies Facility). Data were processed using the Illumina Pipeline Software v1.40.

### ChIP-sequencing data analysis

Sequenced DNA reads (Illumina, 38 bases long reads) were mapped to the human genome (*Homo Sapiens* Genome Reference Consortium Human Build 37 [GRCh37/hg19; Feb. 2009]) using Bowtie with maximally two mismatches and only one hit allowed on the genome. Redundant tags that mapped to the same genomic position and on the same strand were considered PCR duplicates and only one read was kept for analysis. Each lane of the BMAL1 ChIP-Seq yielded 1.6×10^7^ reads, the CLOCK ChIP-Seq 2.1×10^7^, and the CRY1 ChIP-Seq 2.5×10^7^.

### Circadian regulator binding site identification

For each protein separately, BMAL1 or CLOCK or CRY1-bound regions were detected by MACS [32] with the following parameters: shift = 70 bp (BMAL1), 90 bp (CLOCK) and 100 bp (CRY1), bandwidth = 2×shift, genome size = 2.16 Gb, and an input chromatin sample from human 293T cells as control data. This yielded a total of 9459 regions bound by at least one protein. A refined estimate of the binding site location in each of these regions was then obtained by a deconvolution algorithm that models the expected distribution of tags on the positive and negative strands [23]. This was done separately for each protein in order to accurately detect the genomic location of the bound protein. For the rest of the analysis, local maxima in the deconvoluted signal were used as the center positions of the binding sites. The deconvolution methods also provides a goodness of fit score that quantifies whether the plus and minus tag distributions fit the expected patterns [23]. This score was used to reject spurious sites. The binding sites were then attributed a score that combines the enrichment in tag counts in window of ±100 bp around their center compared to input chromatin, as well as the goodness of fit criterion.

The overlaps between the binding sites of the three proteins were computed using the command “intersectBed” from the BedTools suite [33].

Each binding site was annotated with the RefSeq gene having the closest TSS.

Visualization of the ChIP-seq and of the detected binding sites for each protein can be found on the following UCSC genome browser session:

http://genome.ucsc.edu/cgi-bin/hgTracks?hgS_doOtherUser=submit&hgS_otherUserName=laurasymul&hgS_otherUserSessionName=Stefanski_et_al_2013.

### Microarray design

A 60 k customized microarray was designed for 6356 genes, which corresponds to roughly one fourth of the human genome. 1373 genes were assigned to CRBSs, 1503 genes were specifically selected in addition to a set of 3480 random genes. For each gene 10 independent probes were designed to increase reliability of the data.

Two 48-hour time courses were performed as described in the Time courses section and cell pellets were harvested every 3 hours. RNA was purified and samples were labeled with the Two-Color Low Input Quick Labeling kit (Agilent) according to the provided protocol. Samples were labeled in two colors to allow a comparison of RNA amounts from each of the 16 time points (labeled individually with Cyanine-5 (Cy5)) to the average of all time points (labeled together with Cy3). The GEO accession number for the microarray results is GSE57891.

### Microarray analysis

After rejecting probes with too low absolute hybridization signals (less than 20 fold of the median signal of negative control probes), the raw ratio data of the two independent 48 h time-courses was analyzed. Genes with less than two valid.

probes were rejected from the analysis. For each gene, the median ratio of all remaining probes was then used as relative expression measure of one probeset at each time point. For the identification of rhythmic probesets, the Fisher test was used as described [23], [25]. To be stringent the following combined filters were used: the Fisher test p-value needed to pass a threshold of a FDR (False Discovery Rate) [34] of 20% in at least one of the two arrays, and of 50% in both arrays; the amplitude needed to be higher than 0.25 in log2 scale on both arrays, which corresponds to a 1.2-fold change from peak to trough; given that the signal for one gene consisted of the median of at least 3 probes and at most 10 probes, sufficient correlation between these probes was ensured by requiring that the temporal average of the standard deviation between the probes does not exceed 33% of the amplitude of the median signal; the phase of a given gene needed to be comparable in both arrays, allowing for a deviation of ±3 h between both arrays. Together, this analysis yielded a set of 118 robustly rhythmic transcripts.

### Real-time bioluminescence monitoring

30,000 cells/well were split into a 96-well plate the day before the experiment. Before measuring the luminescence, medium was exchanged to DMEM (w/o Phenolred) supplemented with 10% FCS, Hepes (25 mM), luciferin (0.125 µM; P.J.K), Penicillin/Streptomycin. Bioluminescence was recorded every 30 min at 37°C for at least 6 days with an EnVision Xcite Multilabel Reader (Perkin Elmer) and analyzed by the MultiCycle software (Actimetrics). All chemicals were obtained from PAA, if not indicated differently.

### Cell lines

U2OS cells (ATCC # HTB-96) and the thereof derived U2OS *BMAL-luc* cells were described previously [27]. Stable cell lines expressing the promoter-luciferase constructs were generated using retroviral transduction with the pV Pack Eco System (Agilent) according to the manufacturer’s protocol. For lentiviral transduction a modified U2OS cell line, which overexpresses the murine MCAT receptor, U2OS-mcat, had been engineered using selection with the antibiotic zeocin. After transduction single cell clones were sorted using the FACSAria (BD Bioscience) and grown in tissue culture. The clones were then selected for luminescence using the EnVision Xcite system (PerkinElmer). Promoter-luciferase constructs are described in the Supporting information.

### Plasmids and oligonucleotides

Promoter-luciferase constructs were bought from SwitchGear Genomics for *ATG3* (product ID 108396), *EIF5A2 (product ID* 106995), *SCN5A (product ID* 109730), and *GAPDH (product ID* 121624). They harbor roughly 1 kb upstream genomic sequences of respected genes and contain the ChIpped CRBSs: (*ATG3* 113763005-113763997 bp (- strand; chr3), *EIF5A2* 172109014-172109967 bp (- strand; chr3), *GAPDH* 6513163-6514226 bp (+ strand; chr12), *SCN5A* 38665940-38666935 bp (- strand; chr3)). The fragments are coupled to the destabilized luciferase LUC2P from Promega. The promoter for *PER2* (−848 bp to +204 bp from TSS) was amplified from U2OS genomic DNA and cloned into the pGL4.20 vector (Promega), which expresses *luc2*. All promoter-luciferase constructs were cloned into a pSIN-derived vector for retroviral transduction using the restriction enzymes BamHI and MluI. For each reporter, several independent clones with different expression levels were tested to exclude possible insertional effects.

For qRT-PCR quantification of the *BMAL1*, *HPRT1*, *GAPDH*, and *PER1* genes 5′FAM, 3′TAMRA labeled oligos were used. Probes from the Universal Probe Library (Roche) were applied for preRNA and RNA detection of the other genes.

qRT-PCR was performed on the Roche Light Cycler 480 using the TaqMan Gene Expression Master Mix from Applied Biosystems for ChIP-enrichment and RNA measurements and the TaqMan Fast Advanced Master Mix from Life Technologies for pre-mRNA measurements. Sequences of primers and probes for q-RT-PCR are listed in Table 1.

**Table 1:** Sequences of primers and probes for q-RT-PCR.

BMAL1_F	5′-gaagacaacgaaccagacaatgag-3′
BMAL1_R	5′-catgagaatgcagtcgtccaa-3′
BMAL1_probe	5′-tgtaacctcagctgcctcgtcgca-3′
GAPDH_FWD	5′-catcaatggaaatcccatca-3′
GAPDH_REV	5′-gactccacgacgtactcagc-3′
GAPDH_probe	5′-FAM-tccaggagcgagatccctcca-TAMRA-3′
GAPDH_gDNA_F	5′-catcaatggaaatcccatca-3′
GAPDH_gDNA R	5′-ttctccatggtggtgaagac-3′
GAPDH_gDNA_probe	5′-FAM-tactcagcgccagcatcgcc-TAMRA-3′
PER1_F	5′-agacctctcagcctatgagaaagc-3′
PER1_R	5′-cccgacctgccaagattg-3′
PER1_probe	5′-FAM-tggagagcgggactggcatttacg-TAMRA-3′
HPRT1_F	5′-ctggcgtcgtgattagtgat-3′
PER2_F	5′-ctatgtgacagcggcgact-3′
PER2 R	5′-gctgcacgtatcccctca-3′
PER2 probe	UPL-70
NR1D1 F	5′-tgcgtttgttttcattcagc-3′
NR1D1 R	5′-gggcggagctcattatgtaac-3′
NR1D1 probe	UPL-87
HPRT1_R	5′-ctcgagcaagacgttcagtc-3′
HPRT1_probe	5′-FAM-caccctttccaaatcctcagcataatg-TAMRA-3′
ATG3_F	5′-aacagtgaccattgaaaatcacc-3′
ATG3_R	5′-gattttcttcatcacctcagcat-3′
ATG_probe	UPL-87
EIF5A2_F	5′-ttagcctcggcaaaccaa-3′
EIF5A2_R	5′-cttgtgctttagaaatttcctttgt-3′
EIF5A2_probe	UPL-60
SCN5A_FWD	5′-gagcaacttgtcggtgctg-3′
SCN5A_REV	5′-gatttggccagcttgaagac-3′
SCN5A_probe	UPL-12
ATG3_intron_R	5′-cacatggtatcagttggacaaaa-3′
ATG3_intron_probe	UPL-26
BMAL1_intron_F	5′-ctaggcagtacaagaaccaaaagac-3′
BMAL1_intron_R	5′-gatgaatgtagcttttgggtgac-3′
BMAL1_intron_probe	UPL-25
EIF5A2_intron_F	5′-gattacaggcctgagcaagg-3′
EIF5A2_intron_R	5′-tccactagcatcatttcttacctg-3′
EIF5A2_intron_probe	UPL-23
SCN5A_intron_F	5′-gggtgggaggtcaaactgt-3′
SCN5A_intron_R	5′-ctctgtgcatcccctctagc-3′
SCN5A_intron_probe	UPL-87

### Time courses

For temperature entrainment U2OS cells were incubated at alternating 12 h, 33°C and 12 h, 37°C cycles. Medium changes were carried out at the transition from 33°C to 37°C. When used on luciferase reporter cell lines, this method gave similar results to dexamethasone synchronization. For time course A, cells were pre-incubated under temperature entrainment for 4 days. On day 5, 160,000 cells/well were seeded in 35 mm dishes and grown for 5 more days under temperature entrainment until they reached confluence. Growth medium was exchanged directly before the cells were released to constant 37°C.

24 h and 48 h after medium change cells were harvested at 3 h intervals for 24 h. RNA was isolated using the RNeasy kit (Qiagen).

The time course for array B was carried out similarly, with the exception that cells were incubated in two incubators with opposite temperature rhythms and harvested for 12 h on two days.

## Supporting Information

Figure S1 **Circadian binding profile of BMAL1.** (**A**) The specificity of antibodies against BMAL1, CLOCK, and CRY1 was analyzed by Western-blotting of U2OS cell lysates, comparing serum (1∶1000), peptide-blocked serum (serum: 1∶1000, peptide: 10 µg/ml), and affinity-purified antibody.(B) Rhythmic BMAL1 occupancy on the promoters of *PER2* and *REV-ERBα* genes in synchronized U2OS cells. Cell were synchronized with temperature cycles (12 hours of 33°C–12 hours of 37°C) and then released to a constant temperature of 37°C. Samples were collected at four different time points as indicated (n = 3). The CIRCWAVE p values for BMAL1 binding to the PER2 and *REV-ERBα* promoters were 0.0016 and 0.000004 respectively.
(TIFF)

Figure S2 **E-box and double E-box motifs are enriched in BMAL1 and CLOCK binding sites and around the TSS.** (**A**) Upper panel: Sequence logos of E-box motifs enriched in CRBSs. One and two mismatch was allowed. Lower panel: Enrichment of CRBSs containing the indicated E-box motifs (black columns). To control for bias due to sequence composition, the sequences were randomly shuffled and analyzed for E-box motifs (grey columns). At least one of the three E-box variants CACGTG, CATGTG and CAGGTG was found in 43% of the CRBSs. (**B**) Tandem E-boxes with a spacer of 6 bp are enriched in CRBSs. Upper panel: Sequence logos of double E-box motifs. Lower panel: Autocorrelation analysis of E-boxes (23).
(TIFF)

Figure S3 **Temporal expression profiles of 118 rhythmic genes.** RNA levels of temperature entrained U2OS cells were analyzed in 3 h intervals over a period of two days in constant conditions (Materials and methods). For each gene the expression ratios of each time point (0–45 h) relative to the average expression level were calculated and plotted on a log2 scale versus the circadian time (CT). For each gene up to 10 probes were spotted on the arrays. Light blue lines indicate the expression profiles based on individual probes. The black triangles and the fitted dark blue sine curves correspond to the median of the data. Light and dark areas in the background indicate subjective day and night, respectively. Data from array A (left panels) and array B (right panels) is shown.
(PDF)

Figure S4 **Temporal expression profiles of selected rhythmic and non-rhythmic genes.** This figure presents data from array B that is complementary to data from array A shown in Figure 4. Examples are shown that fall in various categories. (**A**) Clock genes with CRBSs. (**B**) Clock-genes without CRBSs. (**C**) Rhythmic genes with CRBSs. (**D**) Rhythmic genes without CRBSs. (**E**) Genes not expressed in rhythmic fashion. *AGAP11* harbors a high scoring binding site for BMAL1, CLOCK, and CRY1, *CHP* has a CRY1 binding site. In both genes the CRBSs were close to the TSS. *CSNK2B* and *KDELR1* do not have a CRBS.
(TIFF)

Figure S5 **mRNA expression phase and correlations with CRBSs.** This figure presents data from array B that is complementary to data from array A shown in Figure 5. Analysis of temporal expression profiles of 5708 expressed genes in two microarray replicates identified 118 common genes with diurnal expression rhythms. (**A**) The amplitude of a gene (n = 5708) was plotted versus the circadian phase. The 118 rhythmic genes are indicated by black symbols. The shade of blue corresponds to the rhythmicity of a gene; light blue = low 24 h-rhythmicity and/or high variation in phase; dark blue = high 24 h-rhythmicity and highly reliable phase. (**B**) The amplitudes of the 118 rhythmic genes are plotted against the phase. Genes with CRBSs are shown with black diamonds, the other rhythmic genes are displayed with gray diamonds. Core circadian clock genes are indicated. (**C**) Phase distribution of rhythmic genes with and without CRBSs.
(TIFF)

Figure S6 **CRBSs in promoters correlate with rhythmic gene expression.** Genes with CRBSs (n = 1373) were divided into 352 genes with at least one CRBS close to the TSS (±1 kb) and 1021 genes with only distant CRBSs. The dark grey columns represent the fractions of rhythmic genes (%) with distant and close CRBSs. The light grey column represents the fraction of rhythmic genes in a pool of 3480 randomly chosen genes without CRBS. Rhythmic genes are significantly enriched in the fraction of genes with close CRBS (p = 9.3×10^−4^, Fisher’s Exact Test).
(TIFF)

Figure S7 **Growth of U2OS cells in presence and absence of rapamycin.** 96-well plates were inoculated with about 25.000 U2OS cells per well in the presence of 200 nM rapamycin (in 1∶100 DMSO) or with DMSO, for control (day 1). The plates were incubated at 37°C and the number of cells were counted at day 1, 2, 4 and 7 (n = 10).
(TIFF)
