# Supplementary figures and images for: Non-Circadian Expression Masking Clock-Driven Weak Transcription Rhythms in U2OS Cells
Source: PLoS One. 2014 Jul 9;9(7):e102238. doi: 10.1371/journal.pone.0102238 (PMC4090172; doi:10.1371/journal.pone.0102238)

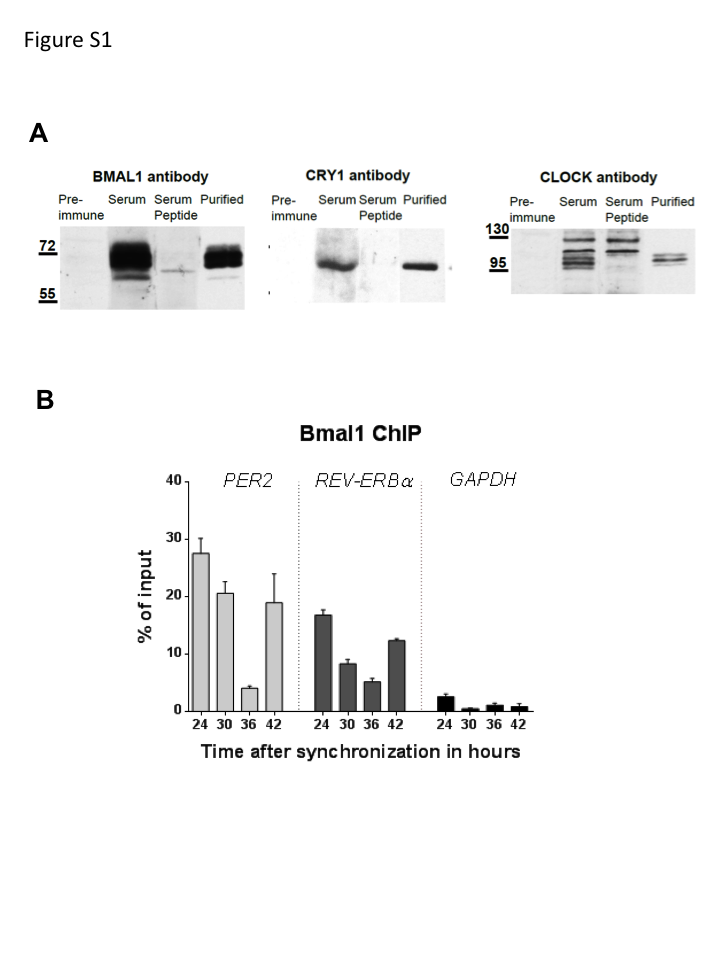

Supplement: Figure S1 — Circadian binding profile of BMAL1. (A) The specificity of antibodies against BMAL1, CLOCK, and CRY1 was analyzed by Western-blotting of U2OS cell lysates, comparing serum (1∶1000), peptide-blocked serum (serum: 1∶1000, peptide: 10 µg/ml), and affinity-purified antibody.(B) Rhythmic BMAL1 occupancy on the promoters of PER2 and REV-ERBα genes in synchronized U2OS cells. Cell were synchronized with temperature cycles (12 hours of 33°C–12 hours of 37°C) and then released to a constant temperature of 37°C. Samples were collected at four different time points as indicated (n = 3). The CIRCWAVE p values for BMAL1 binding to the PER2 and REV-ERBα promoters were 0.0016 and 0.000004 respectively. (TIFF) [file pone.0102238.s001.tiff]

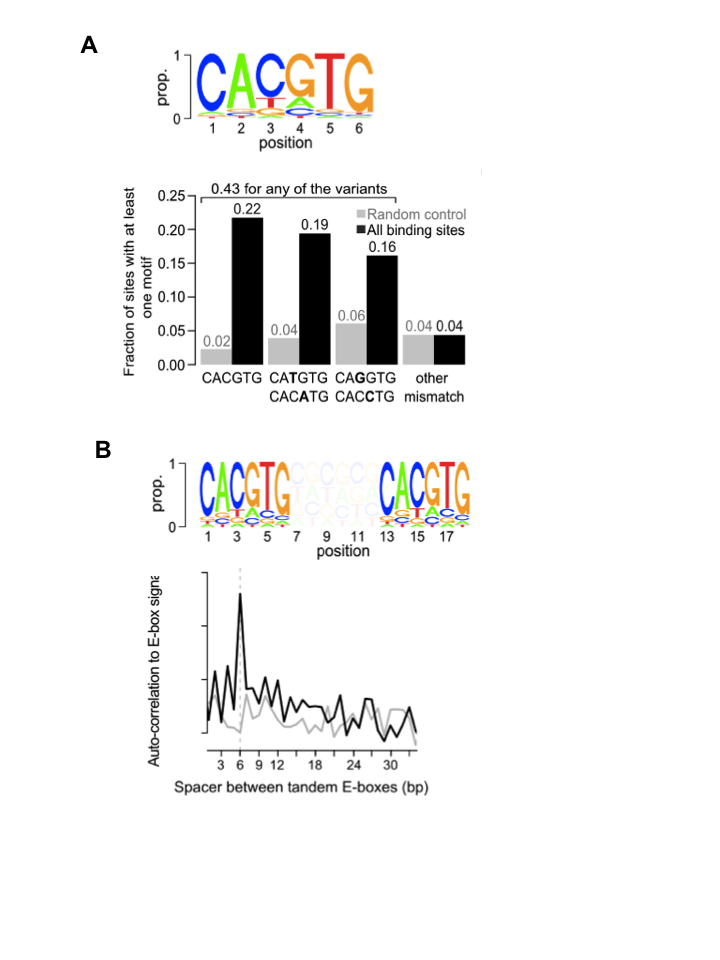

Supplement: Figure S2 — E-box and double E-box motifs are enriched in BMAL1 and CLOCK binding sites and around the TSS. (A) Upper panel: Sequence logos of E-box motifs enriched in CRBSs. One and two mismatch was allowed. Lower panel: Enrichment of CRBSs containing the indicated E-box motifs (black columns). To control for bias due to sequence composition, the sequences were randomly shuffled and analyzed for E-box motifs (grey columns). At least one of the three E-box variants CACGTG, CATGTG and CAGGTG was found in 43% of the CRBSs. (B) Tandem E-boxes with a spacer of 6 bp are enriched in CRBSs. Upper panel: Sequence logos of double E-box motifs. Lower panel: Autocorrelation analysis of E-boxes (23). (TIFF) [file pone.0102238.s002.tiff]

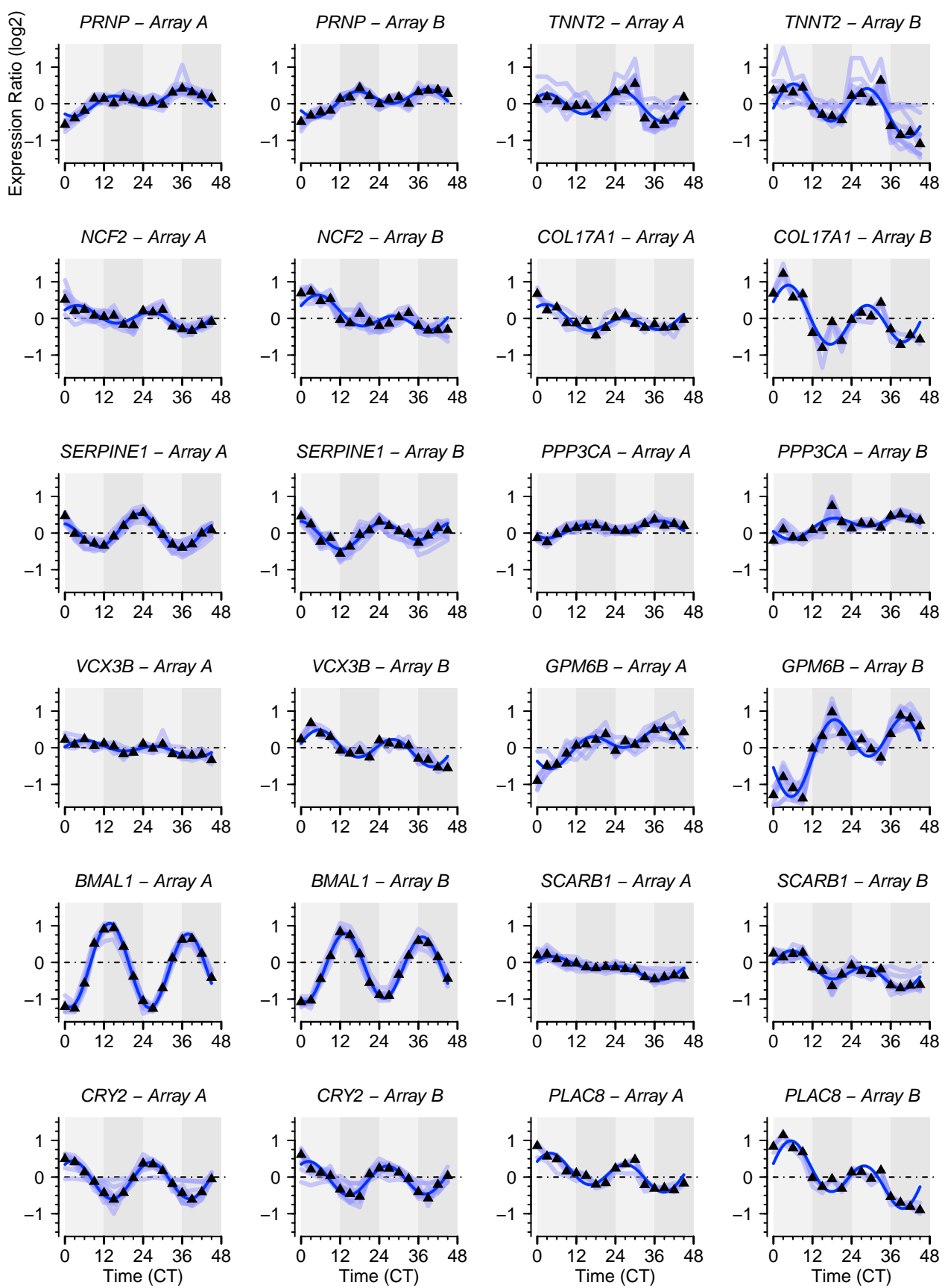

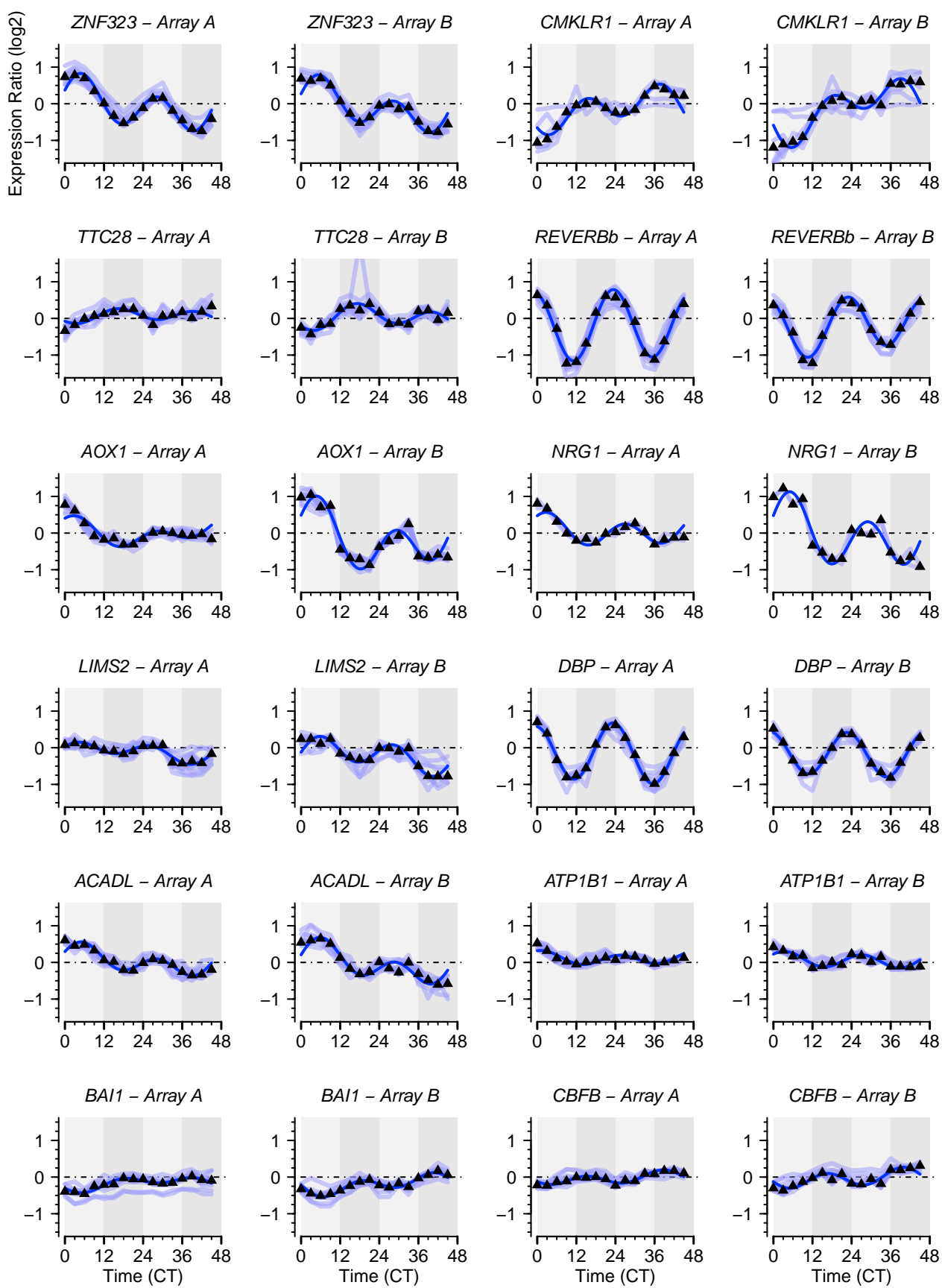

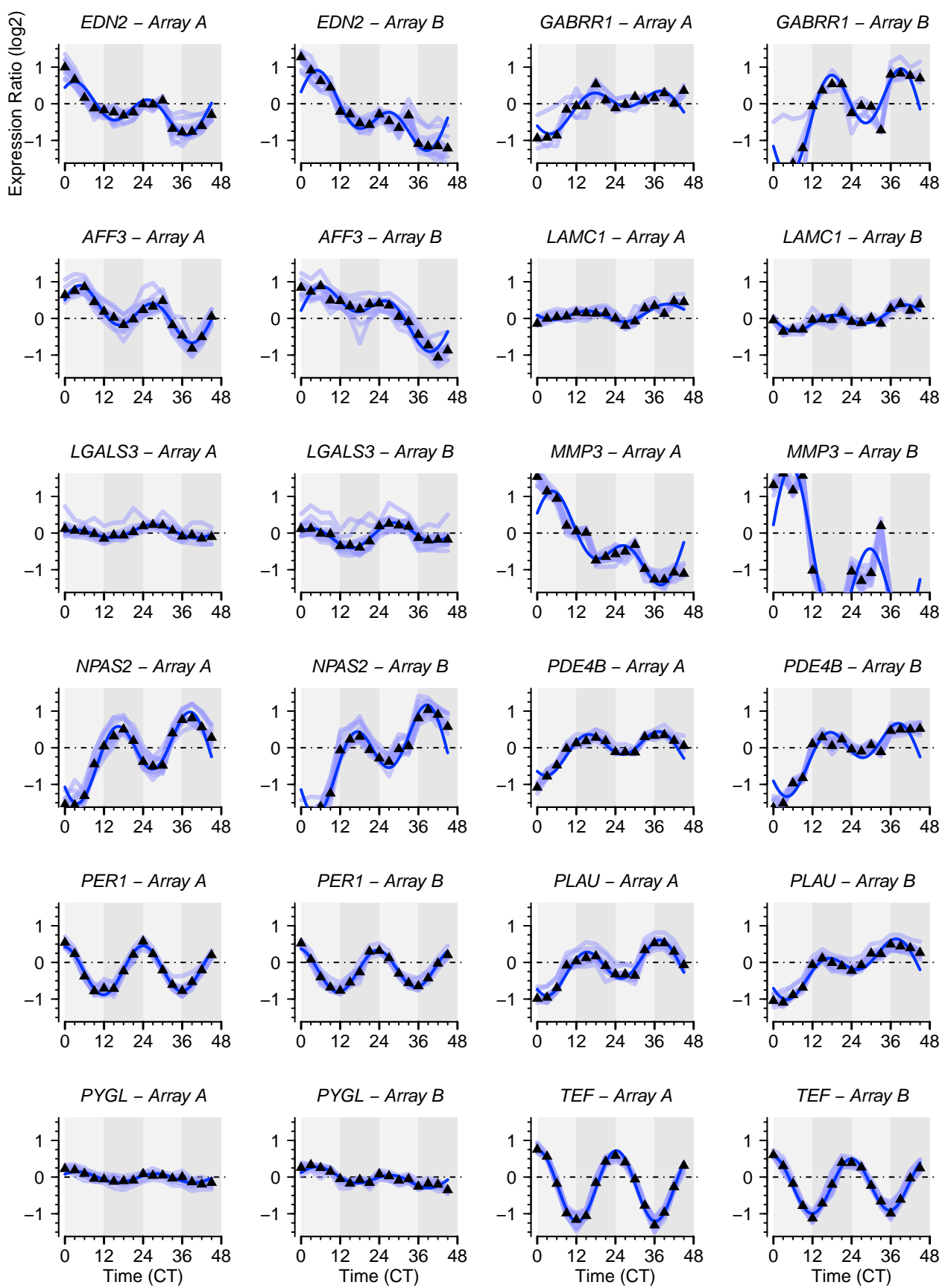

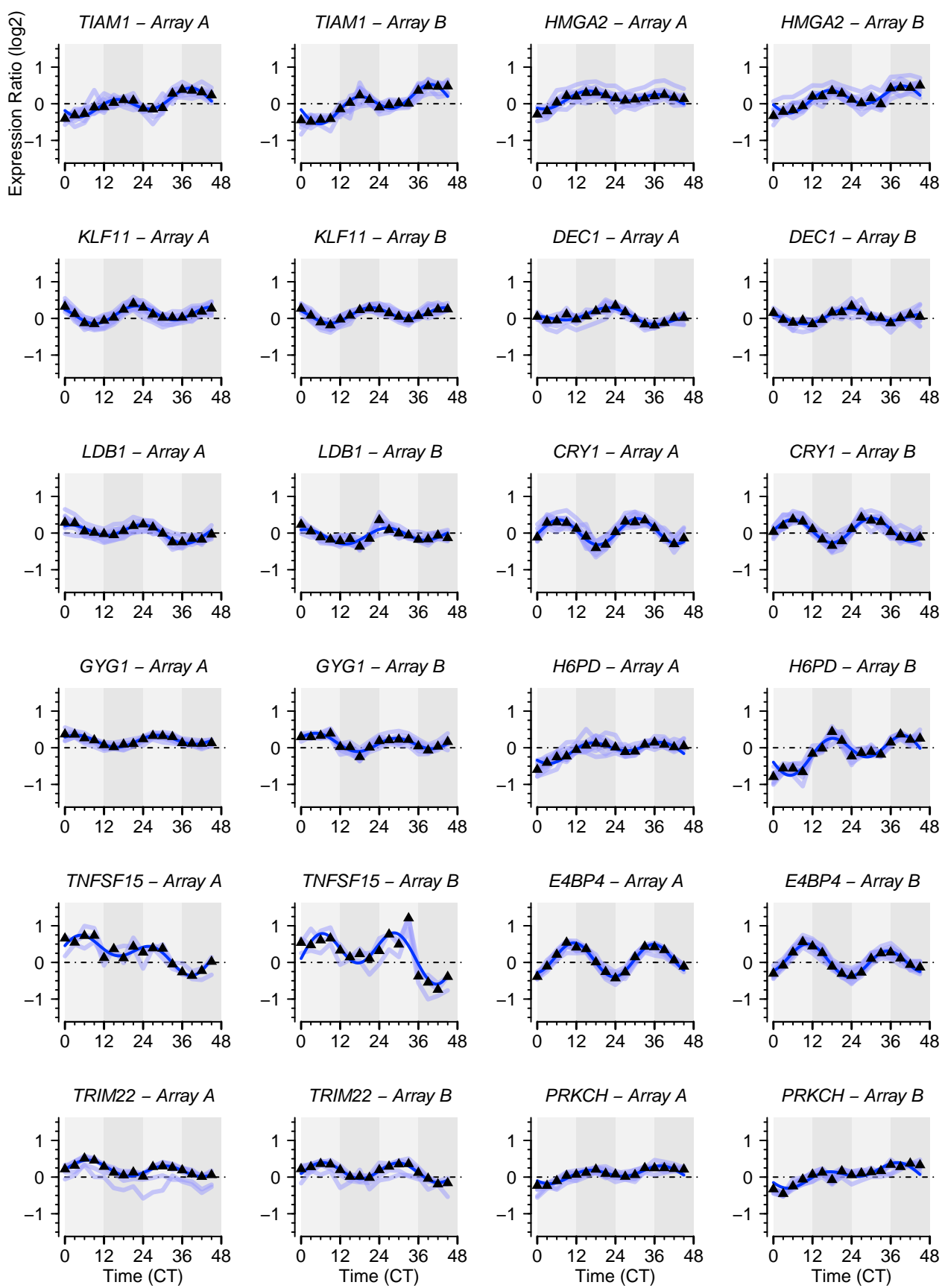

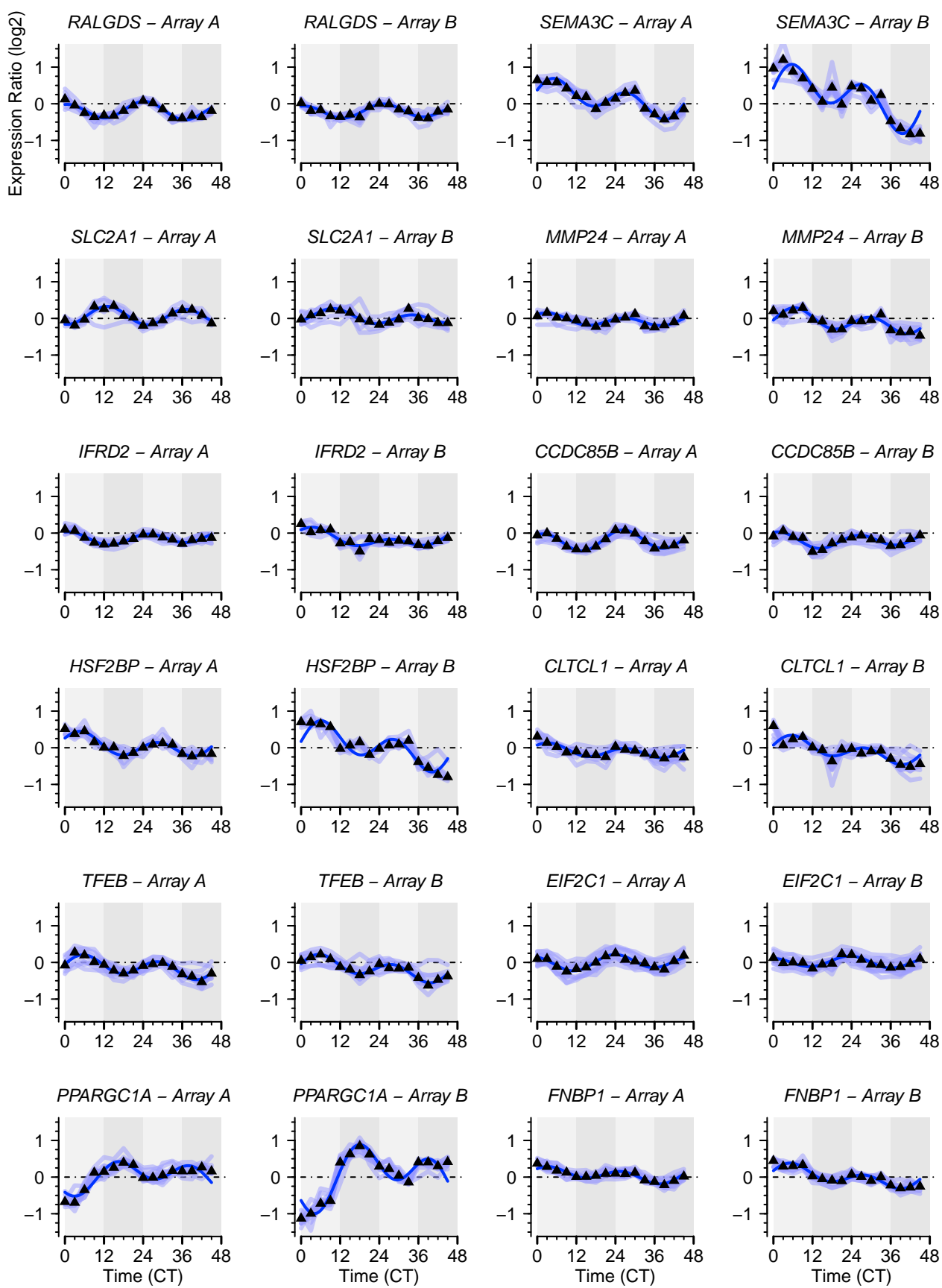

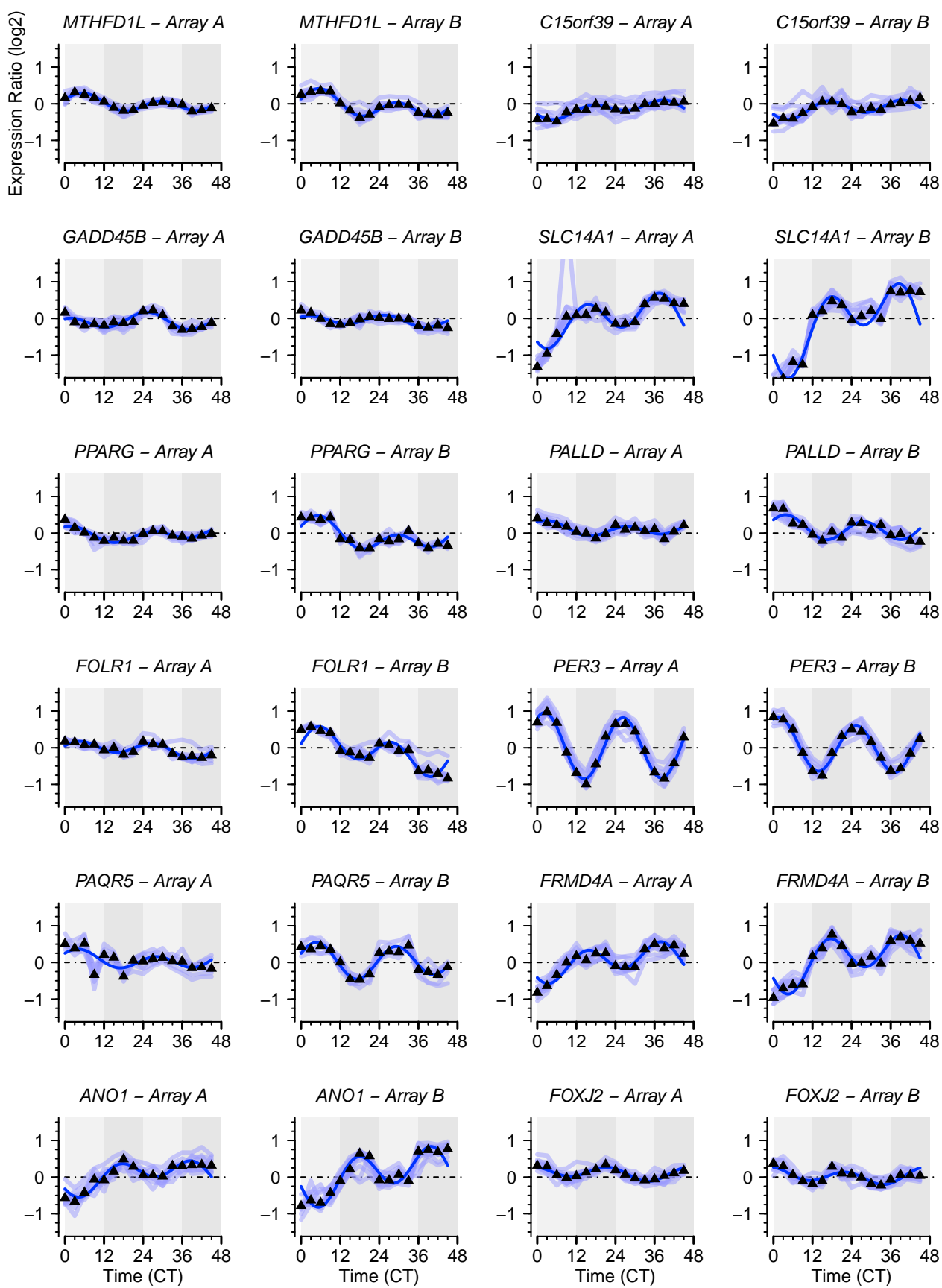

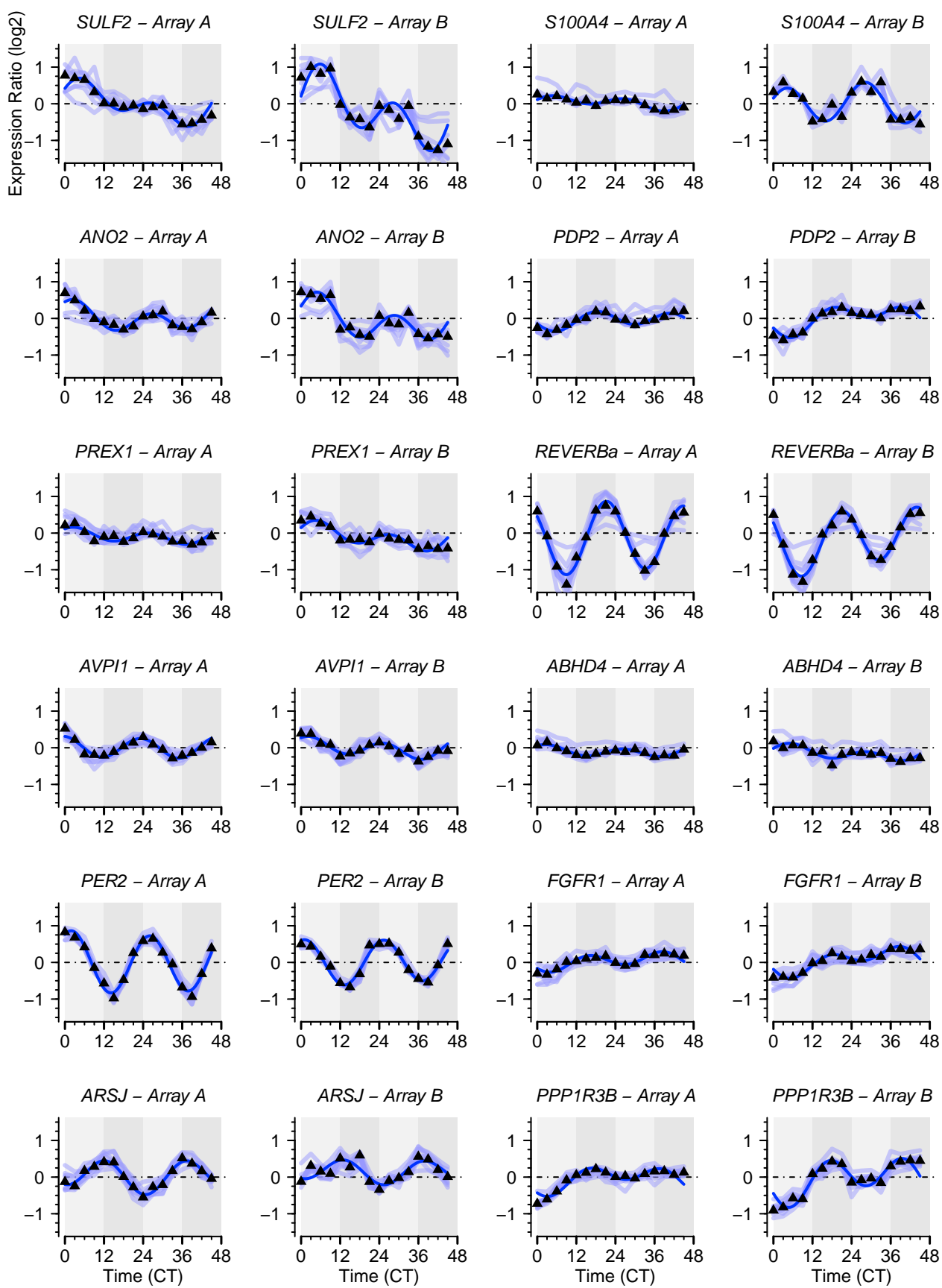

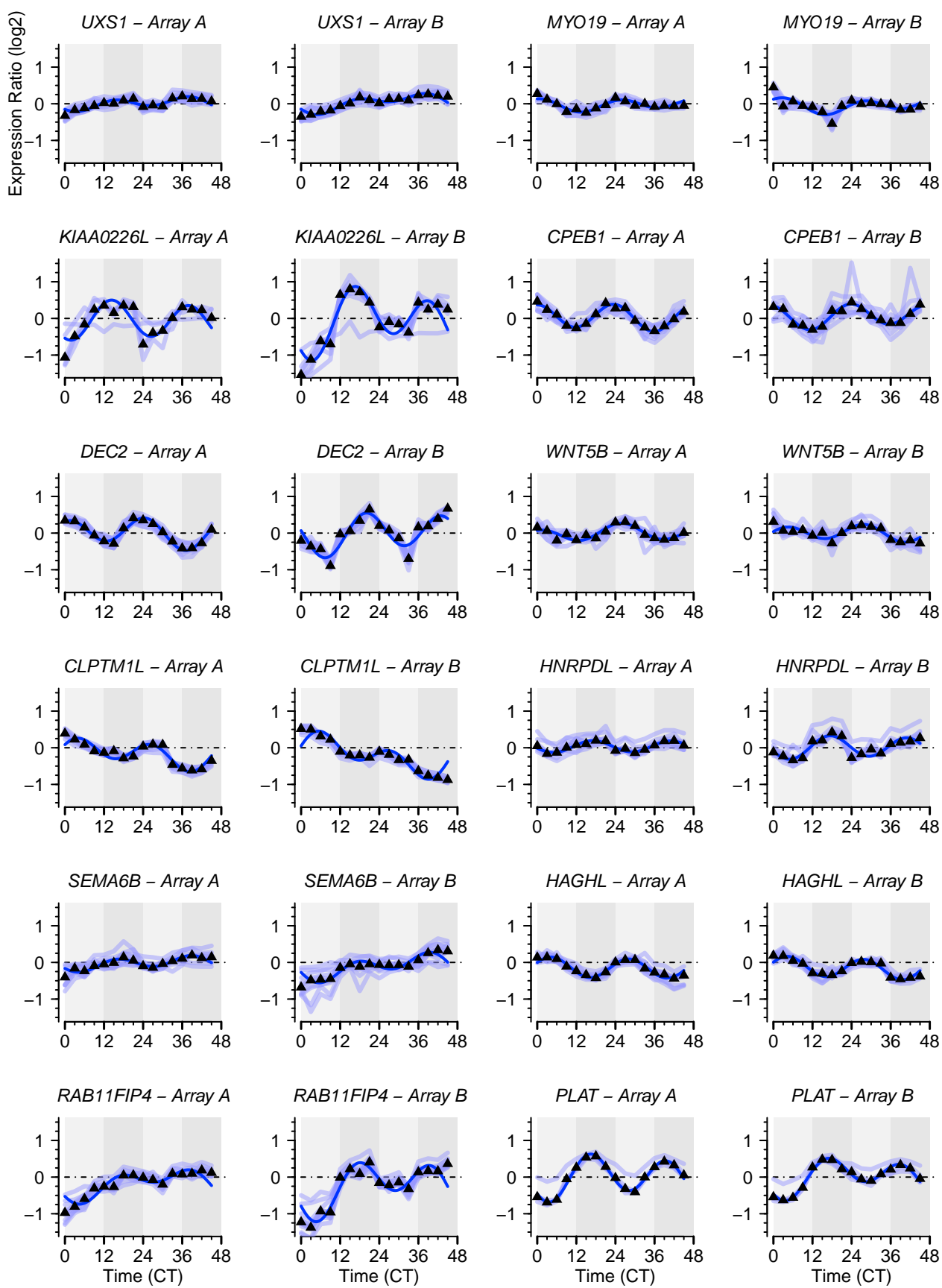

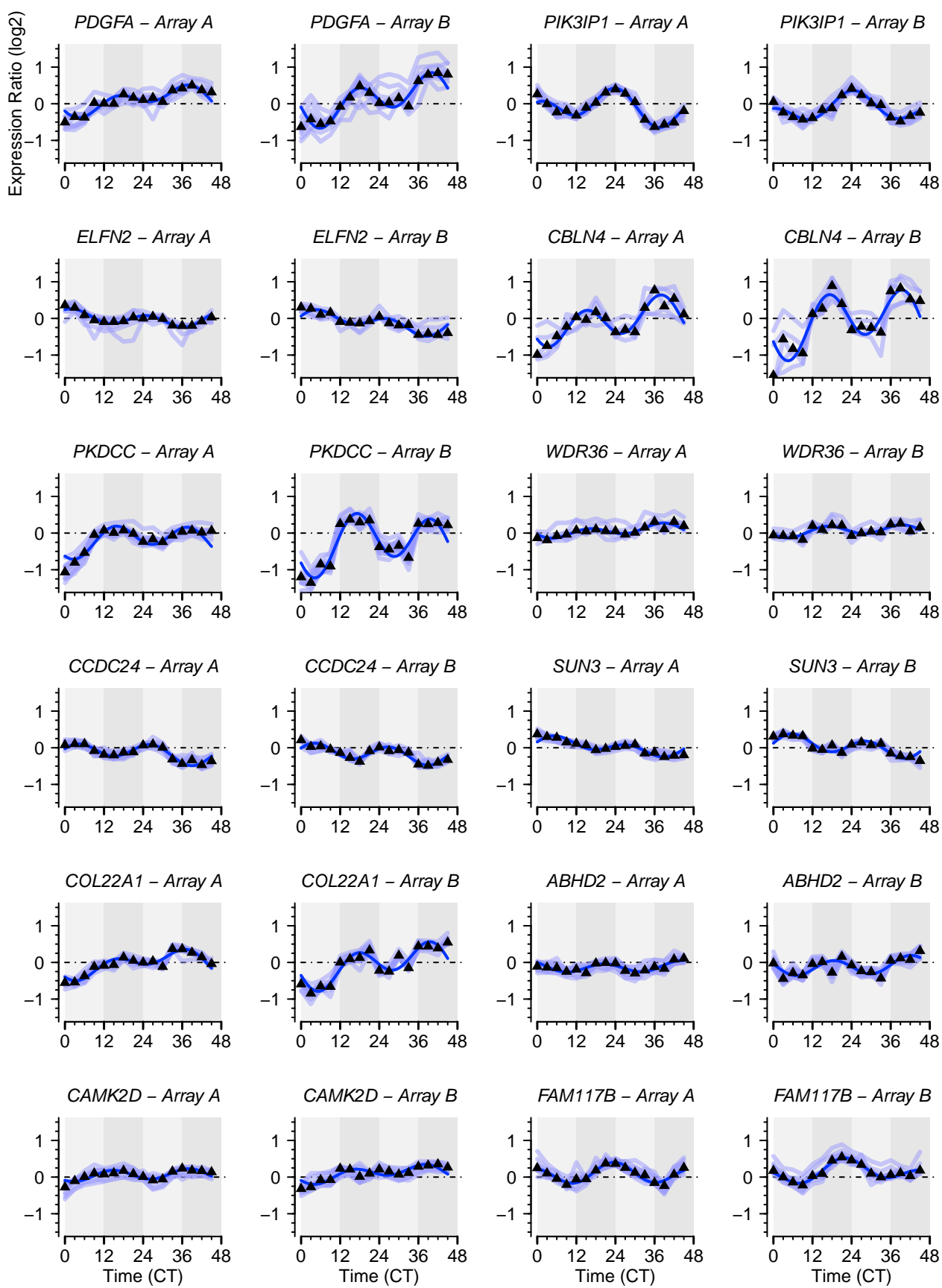

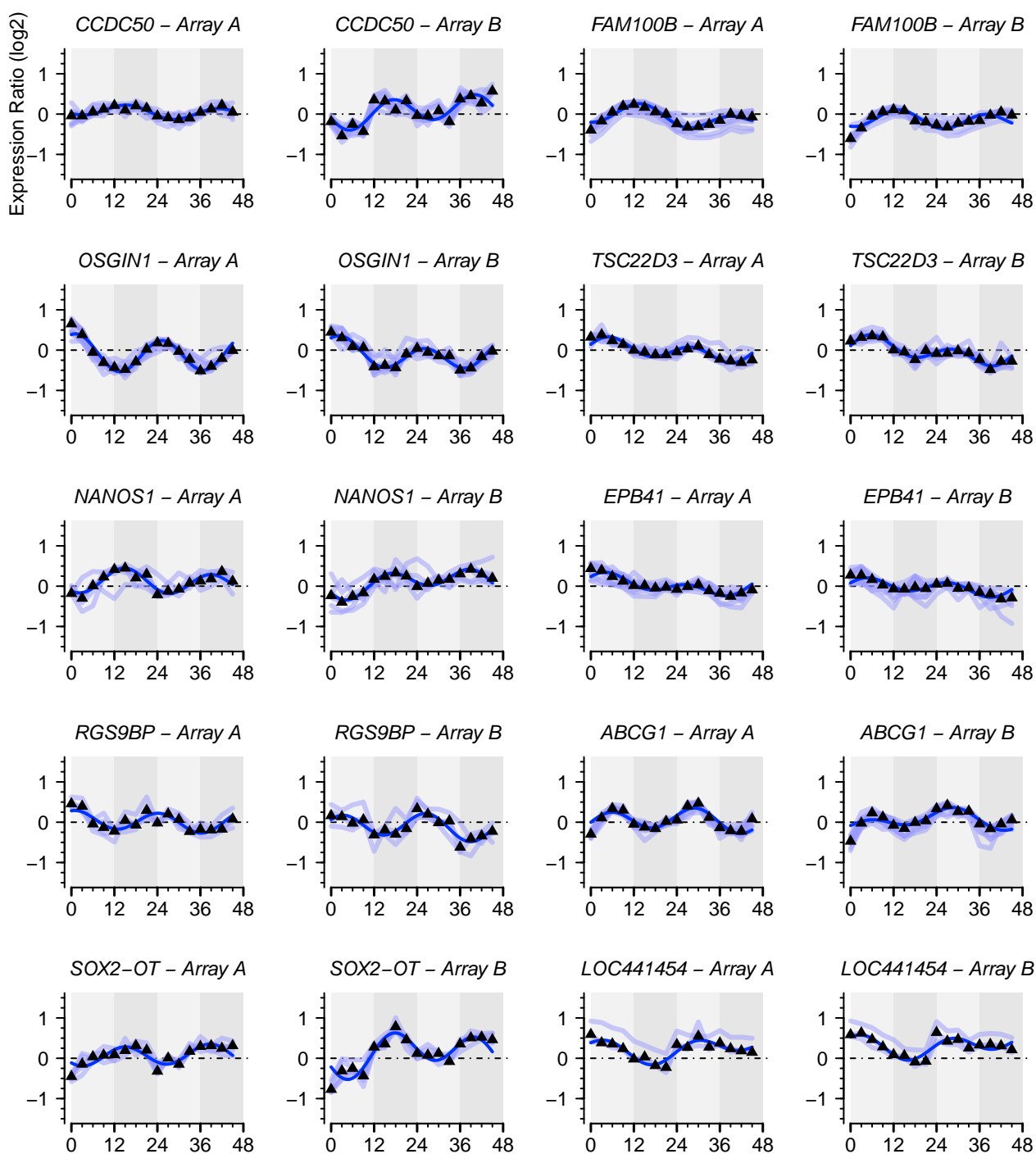

Supplement: Figure S3 — Temporal expression profiles of 118 rhythmic genes. RNA levels of temperature entrained U2OS cells were analyzed in 3 h intervals over a period of two days in constant conditions (Materials and methods). For each gene the expression ratios of each time point (0–45 h) relative to the average expression level were calculated and plotted on a log2 scale versus the circadian time (CT). For each gene up to 10 probes were spotted on the arrays. Light blue lines indicate the expression profiles based on individual probes. The black triangles and the fitted dark blue sine curves correspond to the median of the data. Light and dark areas in the background indicate subjective day and night, respectively. Data from array A (left panels) and array B (right panels) is shown. (PDF) [file pone.0102238.s003.pdf]

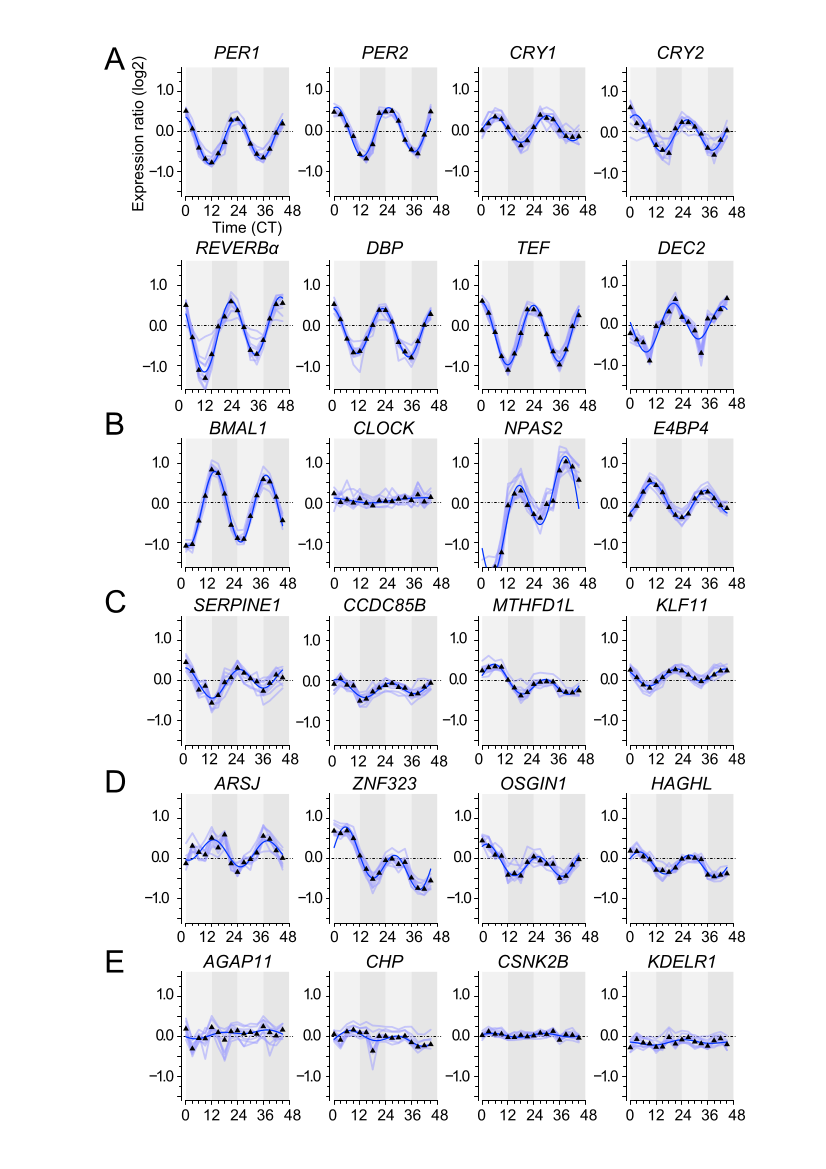

Supplement: Figure S4 — Temporal expression profiles of selected rhythmic and non-rhythmic genes. This figure presents data from array B that is complementary to data from array A shown in Figure 4. Examples are shown that fall in various categories. (A) Clock genes with CRBSs. (B) Clock-genes without CRBSs. (C) Rhythmic genes with CRBSs. (D) Rhythmic genes without CRBSs. (E) Genes not expressed in rhythmic fashion. AGAP11 harbors a high scoring binding site for BMAL1, CLOCK, and CRY1, CHP has a CRY1 binding site. In both genes the CRBSs were close to the TSS. CSNK2B and KDELR1 do not have a CRBS. (TIFF) [file pone.0102238.s004.tiff]

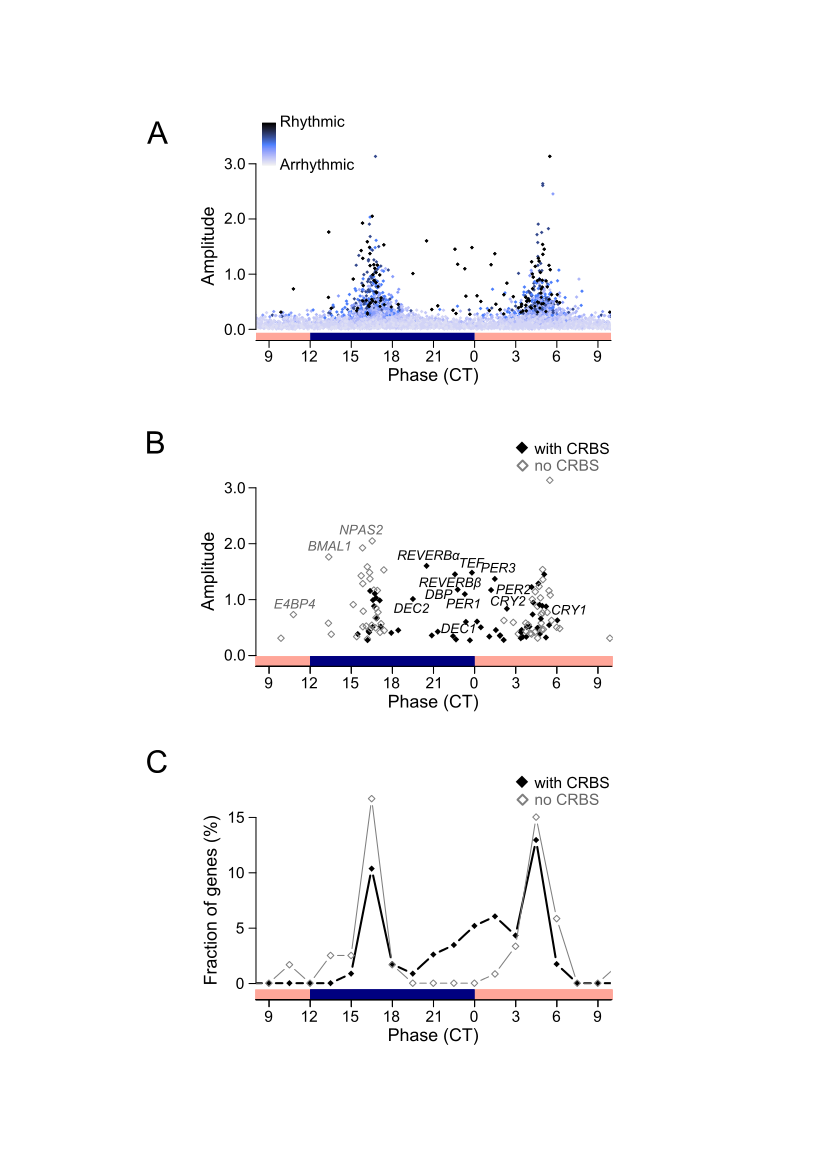

Supplement: Figure S5 — mRNA expression phase and correlations with CRBSs. This figure presents data from array B that is complementary to data from array A shown in Figure 5. Analysis of temporal expression profiles of 5708 expressed genes in two microarray replicates identified 118 common genes with diurnal expression rhythms. (A) The amplitude of a gene (n = 5708) was plotted versus the circadian phase. The 118 rhythmic genes are indicated by black symbols. The shade of blue corresponds to the rhythmicity of a gene; light blue = low 24 h-rhythmicity and/or high variation in phase; dark blue = high 24 h-rhythmicity and highly reliable phase. (B) The amplitudes of the 118 rhythmic genes are plotted against the phase. Genes with CRBSs are shown with black diamonds, the other rhythmic genes are displayed with gray diamonds. Core circadian clock genes are indicated. (C) Phase distribution of rhythmic genes with and without CRBSs. (TIFF) [file pone.0102238.s005.tiff]

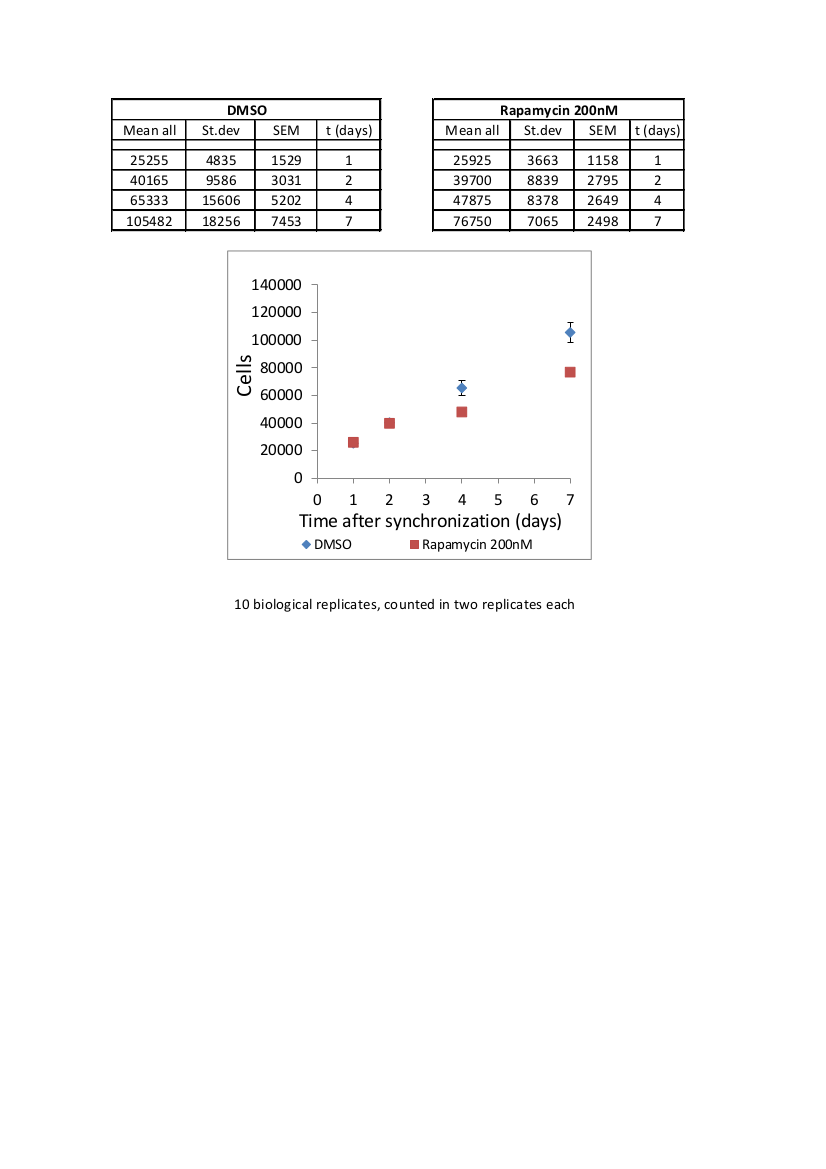

Supplement: Figure S7 — Growth of U2OS cells in presence and absence of rapamycin. 96-well plates were inoculated with about 25.000 U2OS cells per well in the presence of 200 nM rapamycin (in 1∶100 DMSO) or with DMSO, for control (day 1). The plates were incubated at 37°C and the number of cells were counted at day 1, 2, 4 and 7 (n = 10). (TIFF) [file pone.0102238.s007.tiff]
